# Impact of High-Dose Gamma Irradiation on PLA/PBAT Blends Reinforced with Cellulose Nanoparticles from Pineapple Leaves

**DOI:** 10.1021/acsomega.5c06115

**Published:** 2025-08-15

**Authors:** Fernanda Andrade Tigre da Costa, Alain Dufresne, Duclerc Fernandes Parra

**Affiliations:** † Universidade de São Paulo, 119500 Instituto de Pesquisas Energéticas e Nucleares, IPEN−CNEN/SP, Av. Prof. Lineu Prestes, 2242−Cidade Universitária, São Paulo, SP BR 05508-900, Brazil; ‡ Universite Grenoble Alpes, Grenoble INP Pagora (LGP2), 461 Rue de la Papeterie, Gières, Auvergne-Rhône-Alpes FR 38610, France

## Abstract

Polylactic acid (PLA), a widely used biopolymer, faces limitations in melt strength and miscibility with poly­(butylene adipate-*co*-terephthalate) (PBAT), requiring compatibilization strategies. This study uniquely investigates the combined effects of high dose of gamma irradiation (80–150 kGy) and low-aspect-ratio cellulose nanoparticles (CNPs) on PLA/PBAT blends, aiming to enhance compatibility and mechanical performance. Gamma irradiation induced chain scission and radical formation, improving blend compatibility but reducing mechanical properties at high doses due to excessive chain scission. Size exclusion chromatography revealed significant molecular weight reduction from chain scission, with partial recovery at higher doses due to cross-linking or recombination. Scanning electron microscopy (SEM) showed poor CNP dispersion in nonirradiated blends, causing agglomeration and weak interfacial adhesion, while irradiated blends exhibited improved CNP distribution and blend compatibility. Mechanical testing revealed no improvement in tensile strength with CNP addition, as agglomeration and poor dispersion hindered reinforcement, while irradiation-induced brittleness further reduced mechanical performance. Glass transition temperature and thermal stability decreased, as confirmed by differential scanning calorimetry (DSC) and thermogravimetric analysis (TGA), respectively. Rheological analysis showed that CNPs did not significantly enhance viscosity or modulus, likely due to their irregular shape and lack of network formation. Fourier-transform infrared spectroscopy (FTIR) and X-ray diffraction (XRD) highlighted interactions between CNPs and the polymer matrix, with irradiation altering the chemical environment. Contact angle measurements indicated enhanced hydrophilicity with CNP addition in irradiated blends, while biodegradation tests revealed accelerated degradation for irradiated and CNP-reinforced samples. This work innovates by evaluating gamma irradiation and CNPs as compatibilization strategies for PLA/PBAT blends, while identifying limitations for future optimization.

## Graphical abstract

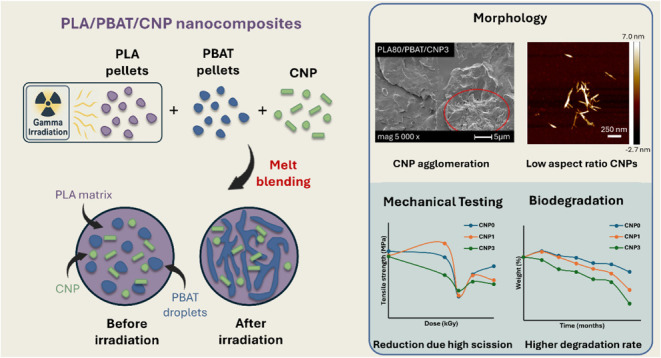

## 1 Introduction

The growing demand for sustainable materials has driven significant research into biodegradable polymers as alternative to conventional petroleum-based plastics. Among these, polylactic acid (PLA) and poly­(butylene adipate-*co*-terephthalate) (PBAT) have emerged as promising candidates due to their complementary properties. PLA, derived from renewable resources such as corn starch, wheat, rice, or sugar cane, is a rigid, high-strength polymer with excellent mechanical properties but limited toughness and slow degradation rates under ambient conditions.^1^ In contrast, PBAT is a flexible, aliphatic-aromatic copolyester known for its good ductility and rapid biodegradability in soil and compost environments.^2^

However, the inherent immiscibility between PLA and PBAT poses challenges for their use in blends, necessitating compatibilization strategies to improve interfacial adhesion and achieve balanced performance.^3^ Similar challenges are observed in other biodegradable material systems, such as PLA/polycaprolactone (PCL) or starch-based blends. PLA/PCL blends often require compatibilization to address the poor miscibility between the rigid PLA and the softer, more ductile PCL,^4–6^ while starch-based blends face issues related to moisture sensitivity and brittleness, often requiring plasticizers or reinforcements to enhance processability and performance.^7^

Compatibilization strategies are typically categorized into physical and chemical methods. Physical methods, such as block copolymers, rely on weak interactions like van der Waals forces or hydrogen bonds to improve interfacial adhesion but may fail under high stress.^8–10^ In contrast, chemical methods use reactive compatibilizers (such as chain extenders, cross-linkers, or ester exchangers) to create covalent bonds between PLA and PBAT, enhancing both adhesion and mechanical strength for durable applications.^11–14^

While conventional compatibilization strategies, such as physical and chemical methods, have been widely explored, gamma irradiation has emerged as a promising technique for compatibilizing immiscible polymer blends, such as PLA/PBAT, by inducing chemical and physical changes at the molecular level.^15–18^ When polymers are exposed to gamma rays, three primary mechanisms occur: chain scission, cross-linking, and branching.^19^ While gamma irradiation offers significant benefits in terms of compatibilization, excessive chain scission at high doses can compromise the material’s mechanical integrity, making it brittle and prone to failure.^18^ Therefore, optimizing the irradiation dose is crucial for achieving a balance between enhanced compatibility and retained material performance.

To further enhance the performance of PLA/PBAT blends, nanocomposites incorporating cellulose nanoparticles (CNPs) have gained attention.^20–23^ For example, Sarul et al.^24^ investigated the addition of cellulose nanocrystals (CNCs), demonstrating their influence on PBAT droplet morphology through scanning electron microscopy (SEM) images and highlighting the formation of a strong CNC network in PLA via rheological tests, though residual solvents limited improvements in ductility and impact strength. Hosseinnezhad et al.^23^ explored PLA/PBAT/cellulose nanofibrils (CNFs) nanocomposites generated in situ, showing that CNFs transformed the PBAT phase from droplets to thinner and longer nanofibers, strengthening interfacial interactions and improving mechanical properties, thermal stability, and shape memory performance without compromising key mechanical parameters. Similarly, Andrade et al.^25^ developed bionanocomposite films reinforced with CNCs derived from sugar cane bagasse fiber, achieving enhanced hydrophobicity, thermal stability, and mechanical properties compared to the neat blend.

CNPs, derived from renewable sources like pineapple leaves through mechanical, chemical, or enzymatic treatments, offer significant advantages due to their high aspect ratio, surface area, and biodegradability. Pineapple leaves, with a high cellulose content of 50.5 ± 1.8%,^26^ are an ideal source for extracting nanocellulose, transforming agricultural waste into valuable materials while addressing environmental challenges posed by current disposal methods such as landfilling and open-air burning, which release toxic compounds and contribute to global warming.^27^ As the second-largest harvested fruit globally, pineapple generates substantial waste, making its leaves a sustainable resource for applications in cellulose recovery, paper production, textiles, and composites.^28^ Nanocellulose exhibits exceptional properties, including low toxicity, biocompatibility, eco-friendliness, high mechanical strength, and modifiability, enabling its use in diverse fields such as packaging, medicine, and electronics.^29^ By converting pineapple waste into nanocellulose, this approach not only minimizes environmental impact but also supports circular economies and the development of sustainable, high-performance materials.

The incorporation of CNPs into PLA/PBAT blends aims to improve mechanical strength. However, achieving uniform dispersion and distribution of nanocellulose in the hydrophobic polymer matrix remains a challenge due to the hydrophilic nature of CNPs, which tend to self-associate via strong interparticle interactions caused by their abundant surface hydroxyl groups.^30,31^ This aggregation becomes more pronounced as particle size decreases, limiting mechanical reinforcement potential.^32^ To address this, Wang et al.^33^ developed an eco-friendly wet-shearing pretreatment method to disperse silane-modified lignocellulose nanofibers (SLCNF) in PLA without organic solvents, enhancing mechanical and thermal properties significantly, while SLCNF boosted PLA crystallinity by 40%. Similarly, Bulota et al.^34^ improved dispersion by acetylating hydroxyl groups of microfibrillated cellulose (MFC) for use in PLA composites, achieving a 70% increase in Young’s modulus and a 60% rise in tensile strength at 20 wt % MFC, with further enhancements in toughness and strain at lower loadings.

Understanding the biodegradation behavior of irradiated PLA/PBAT/CNP nanocomposites is critical for their application in environmentally friendly packaging and disposable products. This study investigates the combined effects of gamma irradiation and CNP reinforcement on the properties of PLA/PBAT blends. Specifically, it explores the structural, thermal, mechanical, rheological, and biodegradation behaviors of these nanocomposites, aiming to optimize their formulation for enhanced sustainability and performance. Additionally, this work aims to evaluate the potential of low-aspect-ratio cellulose nanoparticles (CNPs) as possible reinforcement agents in PLA/PBAT blends, determining whether they can effectively contribute to blend performance or if longer fibers and modified fibers are more suitable. PLA was irradiated using a Cobalt-60 source at various high doses in a multipurpose irradiator to enhance interfacial compatibility by promoting interactions and recombination of macromolecular radicals formed during radiation exposure. By addressing the challenges associated with immiscibility, poor dispersion, and degradation kinetics, this work contributes to advancing the development of the next-generation bioplastics, holding promise as sustainable alternatives for food packaging, agricultural films, and disposable products, where both functionality and environmental impact are critical considerations.

## 2 Materials and Methods

### 2.1 Materials

The biopolymer PLA Ingeo 3D850, with a density of 1.24 g/cm^3^, a melting point between 165 and 180 °C, a melt flow index of 7–9 g/10 min (tested at 210 °C/2.16 kg), a relative viscosity of 4.0 and a d-lactic acid content of 0.5%,^35^ was sourced from NatureWorks LLC, based in Minnetonka, MN. Another material, PBAT Ecoflex C1200 F, characterized by a density of 1.25–1.27 g/cm^3^, a melting temperature of 110–120 °C, and a melt flow index of 2.7–4.9 g/10 min (tested at 190 °C/2.16 kg), was supplied by BASF, located in Florham Park, NJ. The antioxidant Irganox 1010 was acquired from Easy Química LTDA, located in Mogi das Cruzes, SP, Brazil. Pineapple leaves from the Pérola variety (*Ananas comosus* *L. Merril*) were collected from a plantation in Itatiba, São Paulo, Brazil. To obtain cellulose nanoparticles from these leaves, some reagents were utilized: sodium hydroxide (NaOH) obtained from Synth, glacial acetic acid purchased from Control Lab LTDA and 80% sodium chlorite (NaClO_2_) provided by Petra Química.

### 2.2 Sample Preparation

#### 2.2.1 PLA Irradiation

PLA was subjected to irradiation using a multipurpose irradiator located at the Radiation Technology Center (CETER) of IPEN-CNEN/SP. The process utilized a Cobalt-60 source, delivering doses of 80, 100, 120, and 150 kGy, and an average dose rate of 3.4 kGy/h. The irradiation occurred in an air atmosphere at room temperature (25 ± 1 °C), as detailed in a prior study.^18^ The oxidation of PLA was facilitated by the oxidizing conditions during the irradiation process.

#### 2.2.2 Cellulose Nanoparticles (CNPs)

Cellulose extraction from pineapple leaf fibers (PALF) was obtained as reported in our previous study,^26^ involving alkaline treatment and bleaching steps to remove hemicellulose and lignin. Initially, cellulose was isolated through alkaline treatment with 0.2 N NaOH (ratio sample-solution of 1:10) at 100 °C for 90 min, followed by bleaching in a solution of 7 wt % sodium chlorite and 1.4 wt % acetic acid at 80 °C for 4 h (ratio sample-solution of 1:16). The fibers were washed, dried and mechanically ground into cellulose using a tumbler ball mill, model Q298–1 from Quimis Aparelhos Científicos, with an alumina ceramic flask and alumina grinding balls of varying diameters (18 balls of 10 mm, 8 balls of 19 mm, and 5 balls of 50 mm) at 150 rpm for 196 h. The resulting CNPs exhibited an average width of 54 ± 15 nm and an average length of 256 ± 96 nm, as determined by morphological characterization techniques.^26^

#### 2.2.3 Nanocomposites of PLA/PBAT/CNP

PLA/PBAT/CNP nanocomposites were prepared in varying proportions (Table 1). Irganox 1010 was incorporated as an antioxidant agent. The PLA/PBAT blends (50:50 wt/wt) were combined with 1 or 3% CNP using a Haake Rheomex corotational twin-screw extruder, model 332p, from Thermocientific, at 145 °C and 80 rpm, as performed in previous study.^18^ The extruded material was cooled to room temperature, pelletized using a continuous granulation process and injection-molded into test specimens using an AX Plásticos machine, model III.16.AX. Processing conditions included temperatures of 110, 180, 150, and 30 °C across four zones, screw diameter of 16 mm and a length of 384 mm, with operational parameters including a dosing delay of 0.50 s, screw withdrawal speed of 56%, screw turning speed of 95%, cooling time of 5 s, and filling time of 7.5 s, with a steel mold designed according to ASTM D638 – 14 ^36^ for type IV samples. Immediate processing at 145 °C ensured no postradiation oxidation occurred, as this temperature was sufficient to eliminate residual radicals.

**Table 1:** Composition of PLA/PBAT/CNP Specimens

nomenclature	PLA (wt %)	PBAT (wt %)	CNP (wt %)	Irganox (wt %)	irradiation dose in PLA (kGy)
PLA	100	0	0	0	0
PBAT	0	100	0	0	0
PLA/PBAT	49.85	49.85	0	0.3	0
PLA/PBAT/CNP1	49.35	49.35	1	0.3	0
PLA80/PBAT/CNP1	49.35	49.35	1	0.3	80
PLA100/PBAT/CNP1	49.35	49.35	1	0.3	100
PLA120/PBAT/CNP1	49.35	49.35	1	0.3	120
PLA150/PBAT/CNP1	49.35	49.35	1	0.3	150
PLA/PBAT/CNP3	48.35	48.35	3	0.3	0
PLA80/PBAT/CNP3	48.35	48.35	3	0.3	80
PLA100/PBAT/CNP3	48.35	48.35	3	0.3	100
PLA120/PBAT/CNP3	48.35	48.35	3	0.3	120
PLA150/PBAT/CNP3	48.35	48.35	3	0.3	150

### 2.3 Characterizations

#### 2.3.1 Size-Exclusion Chromatography (SEC)

The molecular weight distribution (MWD), poly dispersity index (*D*), and key molecular weight averages (*M_n_*, *M*_w_, *M_z_*, and *M*_p_) were analyzed using size exclusion chromatography (SEC). Viscotek-Malvern GPC MAX VE2001 solvent/sample module paired with a Viscotek TDA 302 triple detector array was utilized. The detector array featured refractive index (RI), low-angle laser light scattering (LALS), right-angle laser light scattering (RALS), intrinsic viscosity differential pressure (IV-DP), and UV detectors (Viscotek model 2501 with a deuterium lamp). Separation was achieved using three PL-GEL mixed-B columns and one PL-GEL precolumn, all kept at 35 °C in a temperature-controlled oven. Tetrahydrofuran (THF) served as the mobile phase, flowing at a rate of 1 mL/min. For sample preparation, approximately 10 mg of PLA, irradiated PLA, or PBAT pellets were dissolved in 5 mL of THF to achieve a concentration of 2 mg/mL. The solutions were continuously stirred at 30 °C for an hour to ensure complete dissolution. Each solution was then filtered through a 0.45 μm CHROMAFIL Xtra H-PTFE syringe filter, discarding the initial 1 mL of filtrate to maintain consistent sample concentration. The remaining solution was collected in vials for injection into the SEC system. Data analysis and MWD profiles were generated using OmniSEC 4.5 software (Viscotek Co.).

#### 2.3.2 Scanning Electron Microscopy (SEM)

The morphology of both irradiated and nonirradiated PLA/PBAT/CNP nanocomposites, was examined using FEI QUANTA-FEG 250 microscope (Thermofischer) operating at an accelerating voltage of 2.5 kV. The samples were cryogenically fractured in liquid nitrogen and subsequently coated with a 5 nm layer of gold/palladium. Fracture surface images of the blends were captured at a magnification of 5.0 kx.

#### 2.3.3 Dynamic Mechanical Analysis (DMA)

Rectangular specimens (6 × 30 × 1 mm^3^) of virgin polymers, blends, and nanocomposites were tested using a Metravib DMA50 dynamic mechanical analyzer in uniaxial tension-mode. Tests were conducted from −60 to 130 °C at a heating rate of 3 °C/min under air flow. Samples were analyzed at a fixed frequency of 1 Hz, with a dynamic strain of 1 × 10^–5^ m and a static force of 0.001 N. To predict the viscoelastic behavior of PLA/PBAT blends and PLA/PBAT/CNP nanocomposites, a percolation-based model adapted from prior studies^37,38^ was applied. This model considers both linear and nonlinear mechanical responses, accounting for interfacial adhesion between PLA and PBAT phases. The blend’s morphology significantly impacts its mechanical properties. The Takayanagi model, a phenomenological approach, was used to predict viscoelastic properties by combining two mixing rules: the Reuss model (series connection) and the Voigt model (parallel connection). A schematic representation of the model is shown in Figure 1, where R and S denote the rigid (PLA) and soft (PBAT) phases, respectively.^39^

**Figure 1:**
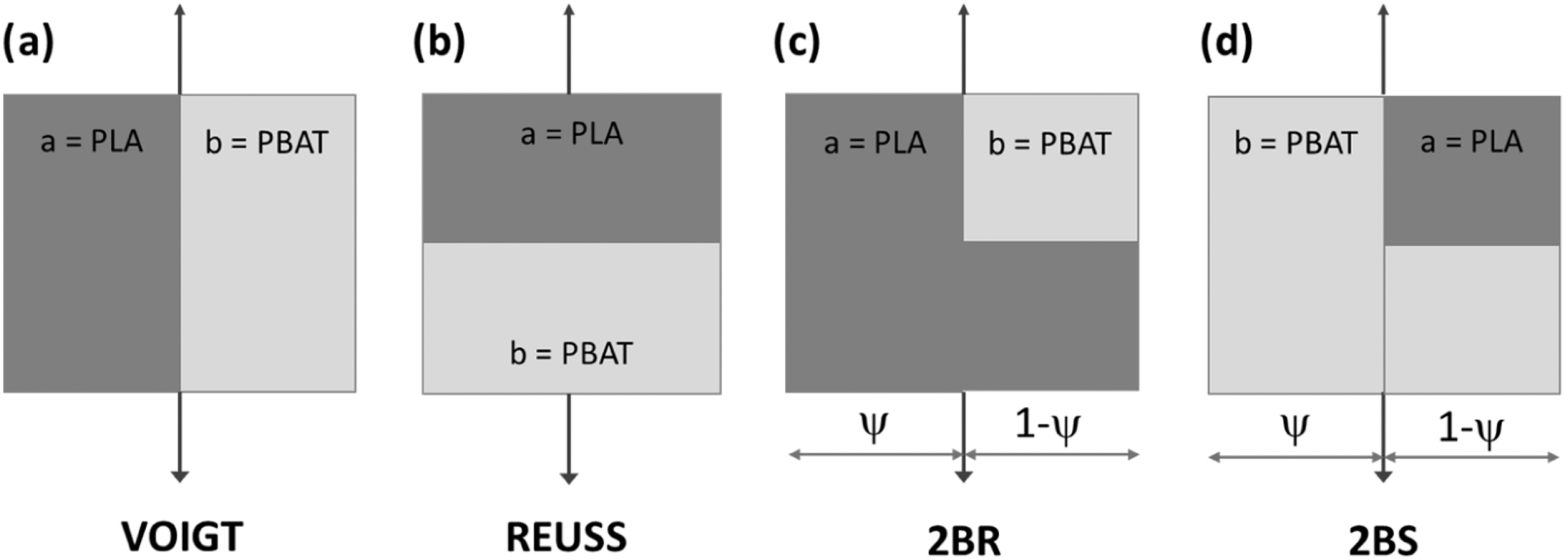
Schematic diagram for the model of: (a) parallel model; (b) series model; (c) “two-branch” R (2BR) model; (d) “two-branch” S (2BS) model.

The model connects the Reuss model in parallel with the percolating volume fractions (ψ_R_ or ψ_S_) of the PLA or PBAT phase, leading to two predictions: the two-branch R (2BR) and two-branch S (2BS) models. Under tensile conditions, the complex tensile modulus (*E*) was predicted using four equations: Voigt (eq 1), Reuss (eq 2), 2BR (eq 3), and 2BS (eq 4). In these equations, σ and ε represent stress and strain, *E*_a_ and *E*_b_ are the experimental moduli of PLA and PBAT, respectively, ϕ_a_ is the volume fraction of PLA (ϕ_a_ = 1 – ϕ_b_, where ϕ_b_ is the PBAT fraction, set at 0.5), and ψ denotes the percolated phase volume fraction. The critical volume fraction (ϕ_ac_) and exponent (*b*) define ψ, as shown in eq 5, with *b* = 0.4 and ϕ_ac_ = 0.25.^37,38^

1 $$E=\frac{\sigma }{\varepsilon }=\phi_{a}E_{a}+\left(1-\phi_{a}\right)E_{b}$$

2 $$\frac{1}{E}=\frac{\varepsilon }{\sigma }=\frac{\phi_{a}}{E_{a}}+\frac{\left(1-\phi_{a}\right)}{E_{b}}$$

3 $$E=\frac{\left(1-2\psi +\psi \phi_{a}\right)E_{a}E_{b}+\left(1-\phi_{a}\right)\psi E_{a}^{2}}{\left(1-\phi_{a}\right)E_{a}+\left(\phi_{a}-\psi \right)E_{b}}$$

4 $$E=\frac{\left(1-2\psi +\psi \phi_{a}\right)E_{b}E_{a}+\left(1-\phi_{a}\right)\psi E_{b}^{2}}{\left(1-\phi_{a}\right)E_{a}+\left(\phi_{a}-\psi \right)E_{b}}$$

5 $$\psi ={\phi_{a}\left(\frac{\phi_{a}-\phi_{\mathrm{ac}}}{1-\phi_{\mathrm{ac}}}\right)}^{b}$$

#### 2.3.4 Differential Scanning Calorimetry (DSC)

The thermal behavior of the samples was analyzed according to ASTM D3418–12 ^40^ using a Mettler Toledo DSC 822e under nitrogen atmosphere (20 mL/min). The analysis involved three stages: first, samples were heated from room temperature to 170 °C at 10 °C/min; then cooled to −30 °C at 10 °C/min to eliminate thermal history; and finally reheated to 170 °C at 10 °C/min after being held at −30 °C for 5 min. Key parameters, including glass transition temperature (*T*_g_), cold crystallization temperature (*T*_cc_), cold crystallization enthalpy (Δ*H*_cc_), melting temperature (*T*_m_), melting enthalpy (Δ*H*_m_), hot crystallization temperature (*T*_hc_), and hot crystallization enthalpy (Δ*H*_hc_), were determined. The degree of crystallinity (*X*_cDSC_) was calculated using eq 6

6 $${X_{c}}_{\mathrm{DSC}}=\frac{{\Delta H}_{m}-{\Delta H}_{\mathrm{cc}}}{{\Delta H}_{m}^{{}^{\circ}}}\times \frac{100}{w}$$

where Δ*H*_m_ is the melting enthalpy, Δ*H*_cc_ is the cold crystallization enthalpy, Δ*H*°_m_ is the melting enthalpy of 100% crystalline material (93 J/g for PLA and 114 J/g for PBAT),^41^ and w is the mass fraction of PLA or PBAT in the blend.

#### 2.3.5 Thermogravimetric Analysis (TGA)

Thermogravimetric analyses were performed in accordance with ASTM E1131–20 ^42^ using a Mettler Toledo TGA/DSC 3+ thermobalance. The samples were heated from room temperature at a heating rate of 10 °C/min up to 600 °C under a nitrogen flow of 50 mL/min, using an open alumina crucible. From the analysis, key parameters were determined: the onset decomposition temperature (*T*_onset_), the temperature corresponding to 5% weight loss (*T*_5%_), the temperature at 50% weight loss (*T*_50%_), and the residue remaining at 600 °C (*R*_600 °C_).

#### 2.3.6 Tensile Tests

Tensile tests were performed on the specimens in accordance with ASTM D638–14 ^36^ standards using an INSTRON universal testing machine, model 5567. The tests were carried out at a cross-head speed of 5 mm/min with a 1 kN load cell. Environmental conditions were controlled at a temperature of 25 ± 5 °C and a relative humidity of 50 ± 5%. Five replicates samples were analyzed per composition.

#### 2.3.7 Rheological Properties

The rheological properties of PLA/PBAT/CNP nanocomposites, both with and without prior irradiation, were evaluated in the molten state using an Anton-Paar Physica MCR301 rheometer equipped with a parallel-plate geometry (*d* = 25 mm). Samples were loaded between the plates and melted at 170 °C, with samples having a thickness of 3.0 mm. Dynamic frequency sweep tests were conducted to determine the viscoelastic properties, using a strain of 1% and an angular frequency range of 150–0.1 rad/s (log scale). The tests provided measurements of complex viscosity (η*), storage modulus (*G*′), and loss modulus (*G*″) in the molten state.

#### 2.3.8 Attenuated Total Reflection Fourier-Transform Infrared Spectroscopy (ATR-FTIR)

The functional groups in the samples were identified using PerkinElmer/Spectrum 65 FTIR spectrometer equipped with a UATR accessory featuring a Diamond/ZnSe crystal (3 reflections). The spectra were recorded over a range of 4000 to 600 cm^–1^, with a resolution of 4 cm^–1^ and 16 scans.

#### 2.3.9 X-ray Diffraction Analysis (XRD)

The degree of crystallinity and peak characteristics were analyzed using a Bruker D2 PHASER diffractometer with Cu Kα radiation (λ = 1.54184 Å) at 30 kV and 10 mA. Diffraction data were collected over a 2θ range of 5–60° at a sweep speed of 0.05°/s. The crystallinity index (*X*_cXRD_) was calculated as the ratio of the crystalline area to the total area using eq 7

7 $${X_{c}}_{\mathrm{XRD}}=\frac{A_{C}}{A_{a}+A_{c}}\times 100$$

where (*A*_c_) represents the crystalline phase area and (*A*_a_) the amorphous phase area. Peak deconvolution and calculations were performed using Fityk software, applying Gaussian functions for the amorphous halo and Voigt functions for the crystalline peaks.

#### 2.3.10 Contact Angle Measurements (CA)

Contact angles were measured using an OCA20 system (DataPhysics Instruments GmbH, Germany) equipped with a Pulnix camera. Distilled water droplets were deposited on hot-pressed films (obtained at 180 °C) with smooth surfaces to evaluate hydrophilicity. Measurements were performed using the SCA20 software module within the first few seconds after deposition. Tests were conducted on dried surfaces at 25 ± 2 °C and 50 ± 5% humidity, with the average contact angle calculated from at least five measurements per sample.

#### 2.3.11 Biodegradation Test

Biodegradation tests were conducted in a controlled natural soil environment following ASTM G160–12.^43^ The soil was prepared by mixing 5 kg each of dry horse manure, sand, and low-clay fertile soil. Hot-pressed film samples (3 × 3 × 0.02 cm^3^) were buried in compartments (6.7 × 6.7 cm^2^) with 2.5 cm of soil above and below each sample. Testing was performed in a controlled room at 23 ± 1 °C and 52 ± 3% relative humidity, with soil moisture maintained at 35 ± 5% and pH adjusted to 6.5–7.5 using elemental sulfur when necessary. Tests were conducted in triplicate for each sample. Mass variation was evaluated monthly for 6 months using eq 8, where *M*_i_ is the initial mass and *M*_f_ is the final mass.

8 $$\mathrm{mass}\;\mathrm{change},\%=\frac{M_{i}-M_{f}}{M_{i}}\times 100$$

## 3 Results and Discussion

### 3.1 Size-Exclusion Chromatography (SEC)

Size exclusion chromatography (SEC) was performed on PLA samples exposed to varying doses of gamma irradiation, as well as on PBAT sample. The molecular weight distribution (MWD) curves for both the initial (nonirradiated) and irradiated PLA samples are presented in Figure 2. These curves reveal a pronounced downward shift in the MWD profiles because of gamma irradiation effect. This trend continues with increasing irradiation doses, although the rate of reduction diminishes at higher doses. This behavior is attributed to polymer chain scission induced by the irradiation process, consistent with findings reported by Otaguro et al.^44^ The MWD curve of the initial PLA sample exhibits a small peak in the low molecular weight region. This feature indicates the presence of a fraction with relatively low molecular weight in the sample. The origin of this low molecular weight fraction may be linked to inherent characteristics of the polymer, such as residual oligomers or unreacted monomers from the synthesis process, or minor degradation that could have occurred during sample preparation or storage prior to testing.

**Figure 2:**
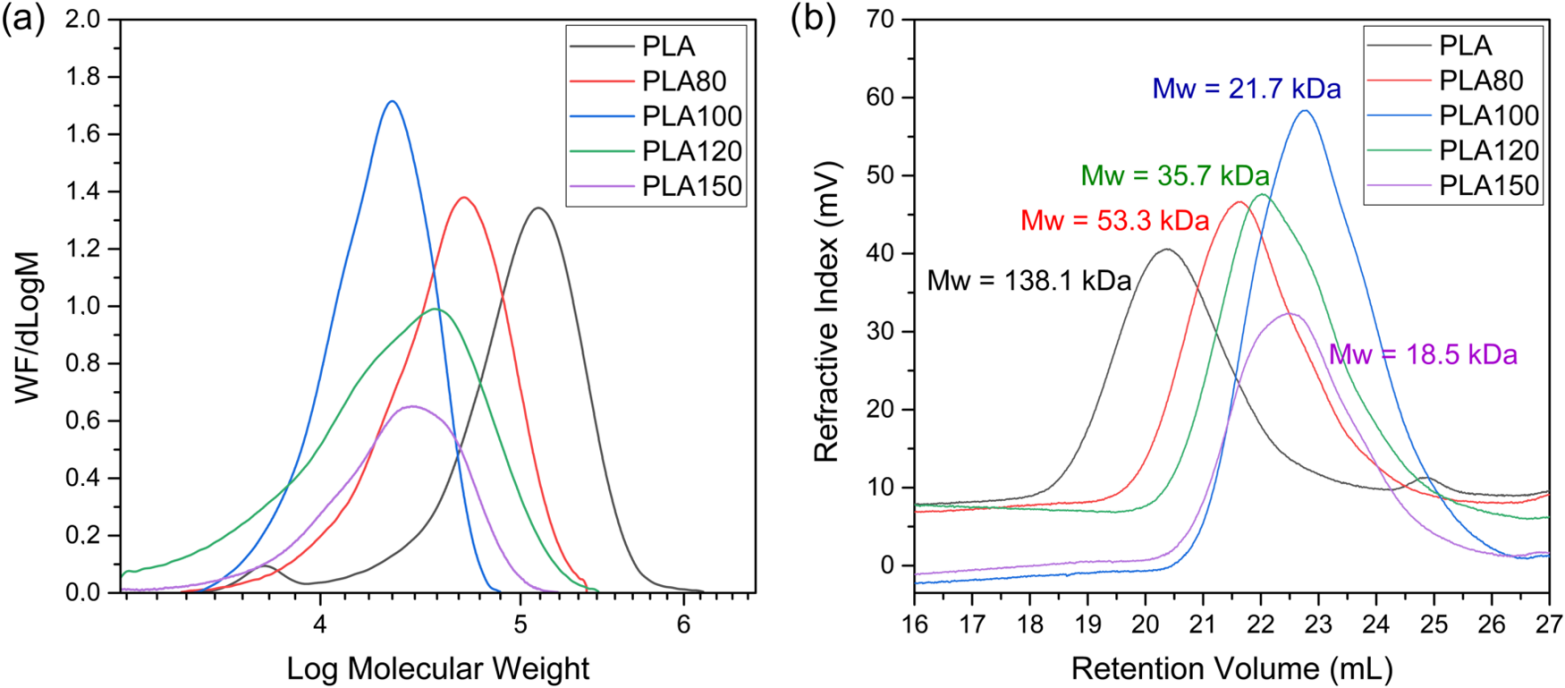
Molar mass distribution of PLA with different irradiation doses: (a) WF/dLog M vs Log M; (b) refractive index vs retention volume.

Further insights into the molecular weight changes are provided in Figure 3 and Table 2, which highlight the significant impact of gamma irradiation on the molecular weight distributions of PLA samples. A clear trend emerges, as the irradiation dose increases, all molecular weight averages, number-average molecular weight (*M_n_*), weight-average molecular weight (*M*_w_), z-average molecular weight (*M_z_*), and peak molecular weight (*M*_p_) decrease. Notably, a sharp decline is observed at 100 kGy due to extensive chain scission. However, at 120 kGy, a slight recovery in molecular weight suggests partial recombination or cross-linking of polymer chains, leading to the formation of larger branched or cross-linked structures. This phenomenon is corroborated by the behavior of the polydispersity index (*D*), which initially decreases due to chain scission but subsequently increases beyond 100 kGy, reflecting broader molecular weight distribution caused by branching or cross-linking events.^44^

**Figure 3:**
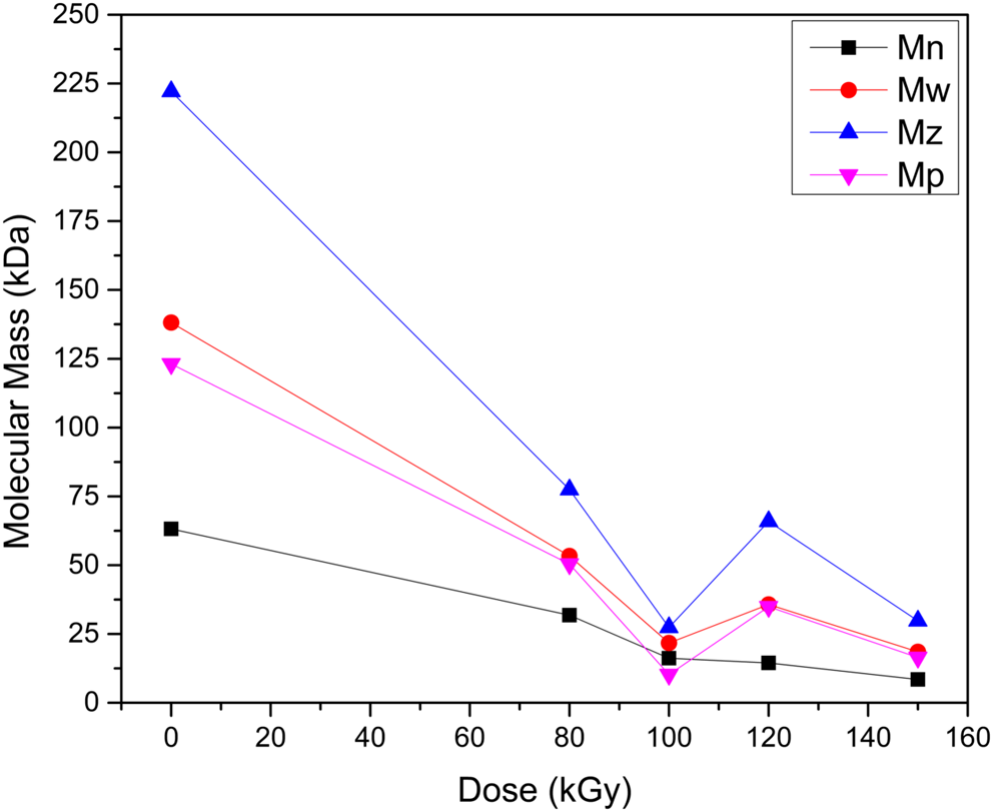
Influence of different doses of gamma irradiation in PLA for various average molecular weight.

**Table 2:** Molecular Weight of PLA Irradiated at Different Doses and PBAT

sample	*M_n_* (kDa)	*M*_w_ (kDa)	*M_z_* (kDa)	*M*_p_ (kDa)	*D* (*M*_w_/*M_n_*)
PLA	63.2	138.1	222.2	123.1	2.2
PLA80	31.8	53.3	77.4	50.4	1.7
PLA100	16.2	21.7	27.4	10.2	1.3
PLA120	14.5	35.7	65.9	34.9	2.5
PLA150	8.5	18.5	29.7	16.5	2.2
PBAT	5.1	54.0	97.7	44.6	10.6

### 3.2 Scanning Electron Microscopy (SEM)

The addition of cellulose nanoparticles (CNPs) to PLA/PBAT blends significantly influences their morphology, as observed in SEM images of the fractured cross-sectional surface (Figure 4). In blends prepared with nonirradiated PLA, the surface remains highly heterogeneous, even with the incorporation and increasing content of CNPs. This heterogeneity indicates limited interaction between nanocellulose and the immiscible polymer blend, likely due to poor dispersion and potential aggregation of CNPs within the matrix. These results are consistent with the phase-separated morphology of PLA/PBAT blends, where PLA acts as the continuous phase and PBAT as the dispersed phase due to their inherent immiscibility.^13^ The lack of compatibility between PLA and PBAT in nonirradiated blends creates a challenging environment for achieving uniform dispersion of CNPs, leading to localized agglomeration and weak interfacial adhesion.

**Figure 4:**
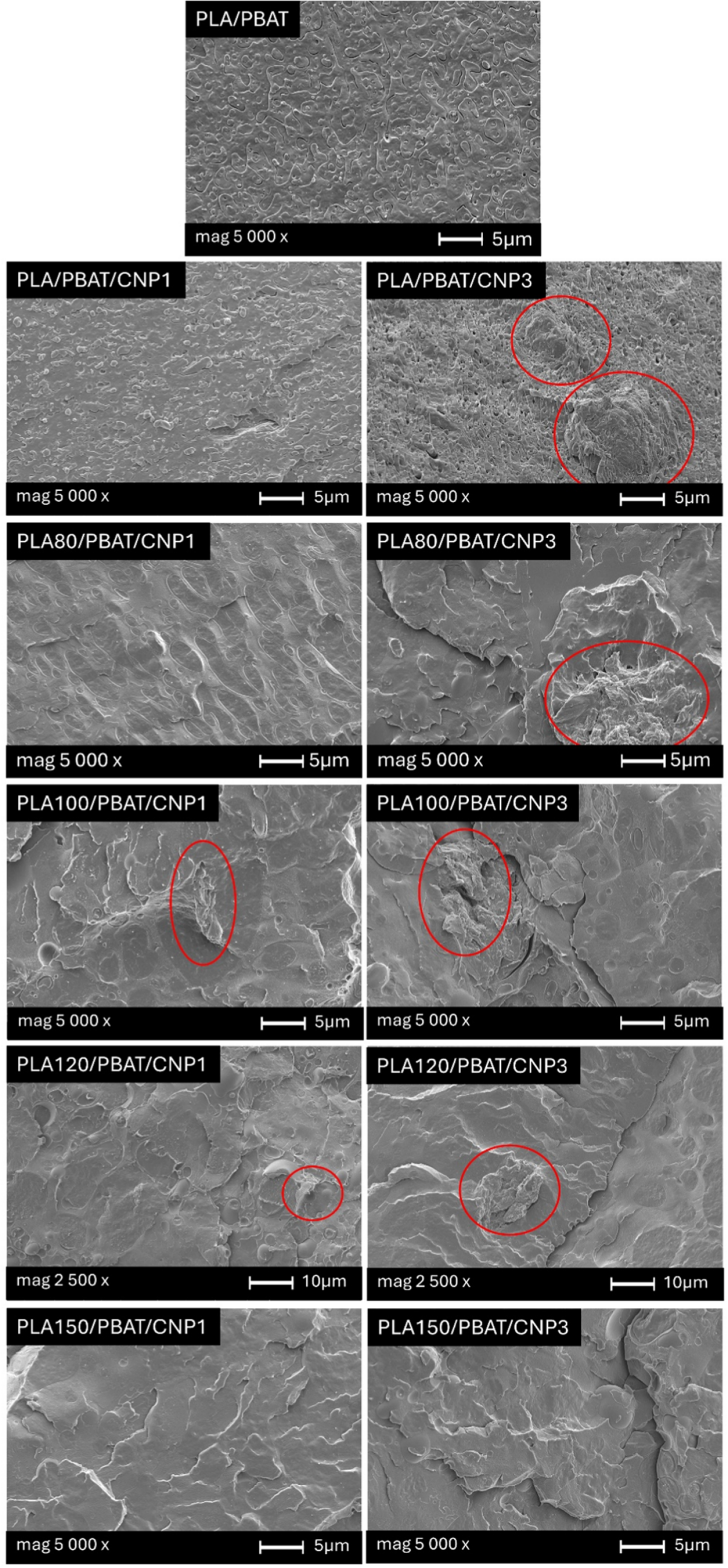
SEM images showing the morphology of PLA/PBAT blend and PLA/PBAT/CNP nanocomposites with 1 and 3% of CNP at different irradiation doses.

The exact localization of CNPs cannot be clearly identified due to the low magnification level. However, as highlighted by the red circles in Figure 4, irregular structures are visible in the samples containing CNPs that do not appear in unfilled blends.^18^ These irregularities likely correspond to CNP aggregates, which cause uneven surface fractures during cryogenic fracture. This uneven distribution suggests that CNPs are not well dispersed throughout the matrix, likely due to incompatibility between hydrophilic, polar CNPs and less polar PLA/PBAT blend, leading to poor dispersion and formation of aggregates. Figure S1 provides Supporting Information about the CNP morphology from AFM analysis, showing that the samples exhibit a low aspect ratio due to excessive processing, which further increases aggregation within the polymer blend matrix. These aggregates create voids at the matrix–filler interface, which act as stress concentration points and negatively impact the composite’s mechanical performance. Consequently, the addition of higher concentrations of CNPs does not enhance the mechanical properties of the PLA/PBAT blend but instead leads to a decline in performance, as shown in Figure 10. This result aligns with findings reported in the literature,^45^ where poorly dispersed microcrystalline cellulose (MCC) similarly reduced performance of PLA/PBAT composites.

In contrast, blends prepared with irradiated PLA exhibit markedly different aspect. As previously reported in an earlier study,^18^ gamma irradiation promotes enhanced miscibility between PLA and PBAT by inducing chemical changes such as chain scission and radical formation. These radicals, confirmed by EPR analysis, facilitate radical combinations between PLA and PBAT during extrusion, improving interfacial interactions and resulting in a more homogeneous blend morphology.^18^ This increased compatibility plays a crucial role in the dispersion and distribution of CNPs within the blend matrix. Specifically, blends containing irradiated PLA show a tendency toward a more uniform interface, particularly with the addition of 3% CNPs, where there is a slight reduction in CNP aggregation. This suggests that the improved miscibility between PLA and PBAT in irradiated blends facilitates dispersion and stronger interfacial adhesion of CNPs, enabling them to interact more effectively with both phases.

### 3.3 Dynamic Mechanical Analysis (DMA)

Dynamic mechanical analysis was used to investigate the effects of gamma irradiation on the glass transition behavior (tan δ peak) and storage modulus (*E*′) of PLA/PBAT blends and their nanocomposites reinforced with CNP. Due to the high fragility of pure irradiated PLA test specimens, DMA analyses could not be performed for these samples. Instead, the study was focused on evaluating the properties of PLA, PBAT, PLA/PBAT blends (both irradiated and nonirradiated), and PLA/PBAT/CNP nanocomposites (with and without irradiation). The tan δ curves for pure PBAT and PLA revealed distinct peaks at −26.4 and 45.8 °C corresponding to their individual glass transition temperatures (*T*_g_), as showed in Figure 5a and summarized in Table 3. For PLA/PBAT blends, the tan δ curves exhibited two distinct peaks, reflecting the coexistence of both PLA and PBAT phases within the blend. This dual-phase behavior confirms the immiscibility of PLA and PBAT in the absence of compatibilization.

**Figure 5:**
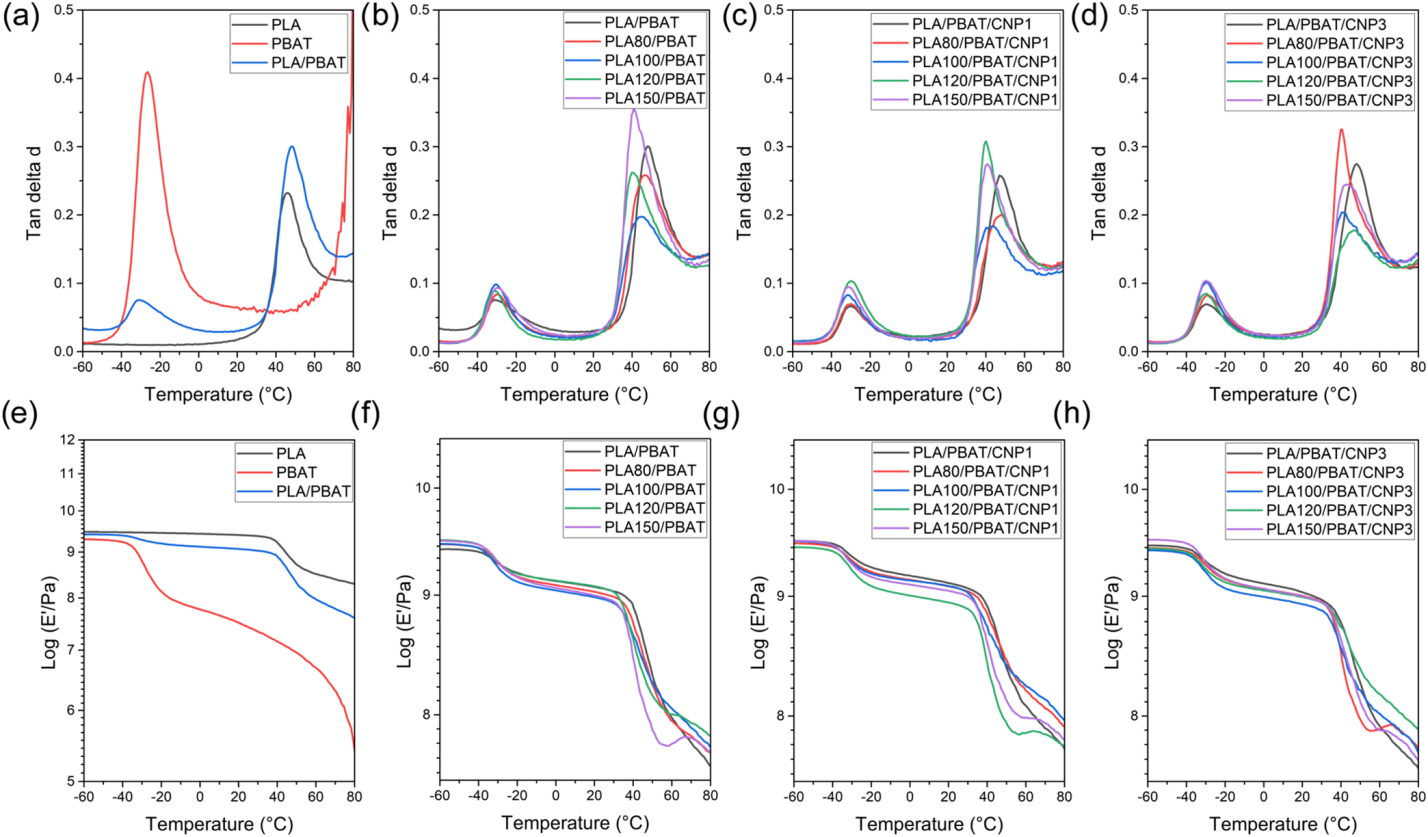
DMA curves of loss factor (tan δ) (a–d) and storage modulus *E*′ (e–h) for: PLA, PBAT and PLA/PBAT blend (a, e); PLA/PBAT blends with different doses of gamma irradiation (b, f); nanocomposites with 1% of CNP (c, g); nanocomposites with 3% of CNP (d, h).

**Table 3:** First and Second Glass Transition Temperatures (*T*_g_) Determined from DMA Curves

sample	*T*_g1_ (°C)	*T*_g2_ (°C)
PLA	-	45.8
PBAT	–26.4	-
PLA/PBAT	–31.0	48.2
PLA80/PBAT	–29.7	46.5
PLA100/PBAT	–30.5	44.7
PLA120/PBAT	–31.2	39.9
PLA150/PBAT	–29.5	40.8
PLA/PBAT/CNP1	–30.3	47.1
PLA80/PBAT/CNP1	–30.3	47.5
PLA100/PBAT/CNP1	–31.6	42.3
PLA120/PBAT/CNP1	–30.1	39.9
PLA150/PBAT/CNP1	–31.2	40.6
PLA/PBAT/CNP3	–29.1	48.2
PLA80/PBAT/CNP3	–28.8	40.3
PLA100/PBAT/CNP3	–29.4	40.6
PLA120/PBAT/CNP3	–30.3	46.4
PLA150/PBAT/CNP3	–29.9	42.8

For blends containing PLA irradiated at varying doses (Figure 5b), a clear trend emerged, the tan δ peak associated with PLA shifted to lower temperatures as the irradiation dose increased. For instance, *T*_g_ of PLA in blends containing PLA irradiated at 150 kGy decreased to 40.8 °C, compared to 48.2 °C for the nonirradiated blend. This reduction in Tg aligns with previous findings, where gamma irradiation-induced chain scission was identified as a contributing factor. Chain scission reduces the molecular weight of PLA, thereby increasing chain mobility and lowering the glass transition temperature.

Gamma irradiation also significantly impacted the storage modulus (*E*′) of PLA/PBAT blends (Figure 5f). The nonirradiated blend exhibited the highest storage modulus at room temperature, while increasing irradiation doses consistently reduced this value. Since the storage modulus reflects the material’s elastic response,^46^ this decrease highlights the weakening effect of gamma irradiation-induced chain scission on the blend’s mechanical integrity.

The influence of CNP incorporation on DMA behavior was further explored in Figure 5c,d,5g,h. The addition of CNP resulted in a slight shift in the tan δ peak corresponding to the PBAT phase toward higher temperatures, indicating an increase in *T*_g_ (*T*_g1_) with the incorporation of CNPs, suggesting potential interactions between the nanofiller and the polymer matrix. In contrast, no significant changes were observed in the *T*_g_ (*T*_g2_) for the PLA phase. The addition of 1% CNP demonstrated a more pronounced enhancement of the PLA/PBAT matrix’s storage modulus, consistent with findings by Arslan et al.^47^ This improvement was particularly evident at lower temperatures. Specifically, the storage modulus of 1% CNP nanocomposite exceeded that of neat PLA/PBAT blend up to approximately 46 °C, beyond which the neat blend exhibited higher values. In contrast, the addition of 3% CNP led to a smaller increase in storage modulus at lower temperatures, with the neat blend overcoming the nanocomposite below −13 °C. This behavior may be attributed to agglomeration of CNPs at higher loadings, which can hinder effective stress transfer and reduce the material’s overall modulus. As expected, increasing the irradiation dose further decrease the storage modulus across the entire temperature range, likely due to the increased brittleness caused by chain scission.

To predict the viscoelastic behavior of PLA/PBAT blends, experimental DMA data were compared with theoretical models. Initially, the data were analyzed using the Reuss model (series configuration) and the Voigt model (parallel configuration), as showed in Figure 6a,b. The results indicated that the experimental data aligned more closely with the Voigt model, suggesting that a parallel arrangement better represents the blend’s morphology. Subsequently, the “two-branch” R (2BR) and “two-branch” S (2BS) models were applied (Figure 6c,6d). The experimental data correlated well with the 2BR model, confirming the blend’s morphology, where PLA serves as the continuous matrix phase and PBAT as the dispersed phase.

**Figure 6:**
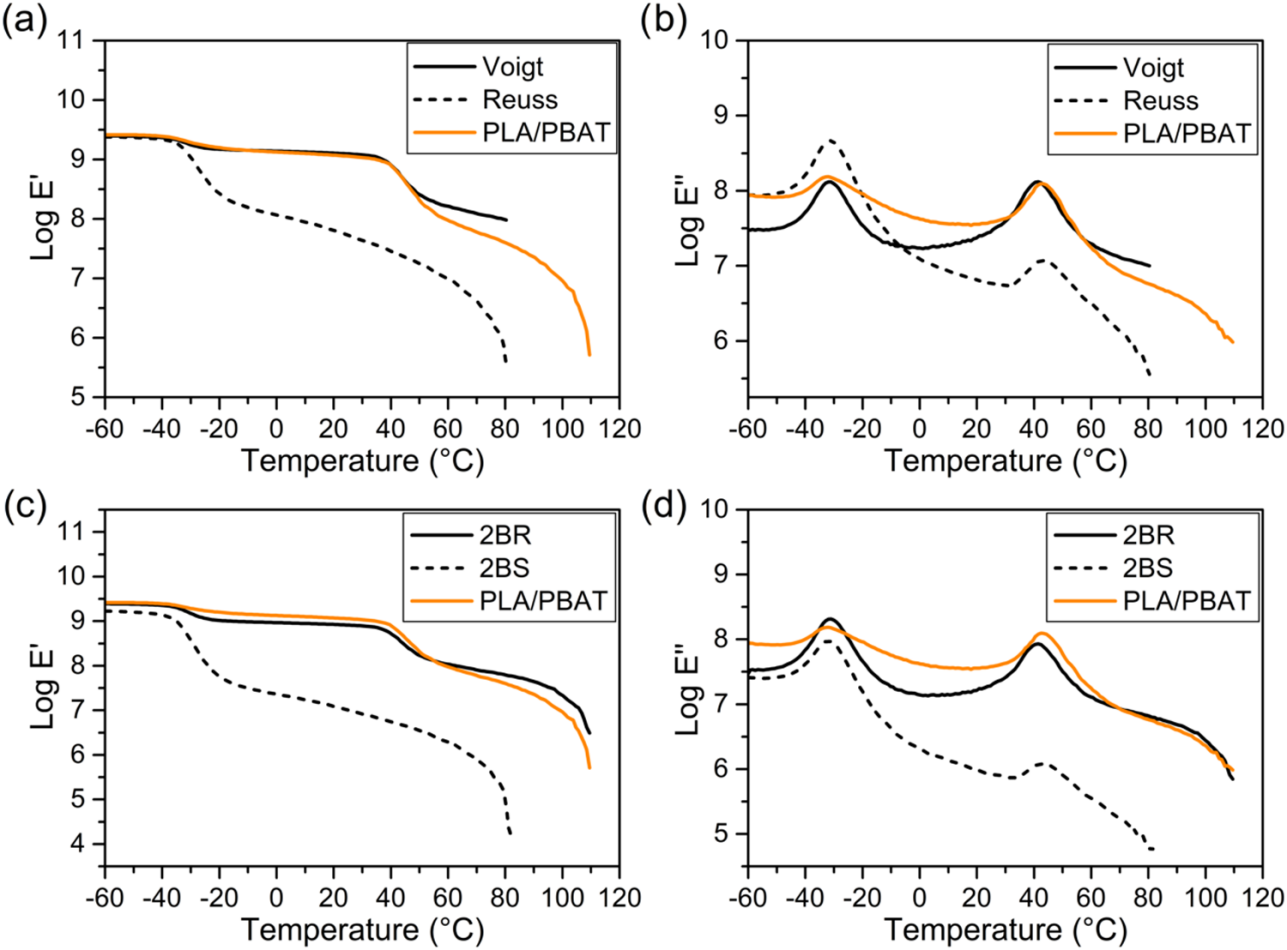
Experimental data for PLA/PBAT blend and predicted data of storage tensile modulus (Log *E*′) and loss tensile modulus (Log *E*″) from (a, b) Voigt and Reuss models; (c, d) “two-branch” R (2BR) and “two-branch” S (2BS) models.

Figure 7 shows experimental and predicted *E*′ and E″ data for blends containing PLA irradiated at the maximum applied dose of 150 kGy, and with cellulose nanoparticle contents of 1 and 3%. It is worth noting that for predicted data the experimental data corresponding to unirradiated blends were used, as these data cannot be determined for irradiated samples because of their brittleness. All specimens exhibited characteristics similar to those of the neat PLA/PBAT blend. This suggests that neither gamma irradiation nor the incorporation of cellulose nanoparticles significantly alters the interaction between the matrix phase (PLA) and the dispersed phase (PBAT) within the blend. Despite the reduction in molecular weight and increased chain mobility induced by gamma irradiation, the fundamental morphology and phase interactions of the blend remain largely unaffected. This indicates that while irradiation and CNPs influence certain properties, such as mechanical performance or thermal behavior, they do not substantially modify the interfacial dynamics between PLA and PBAT in the blend system.

**Figure 7:**
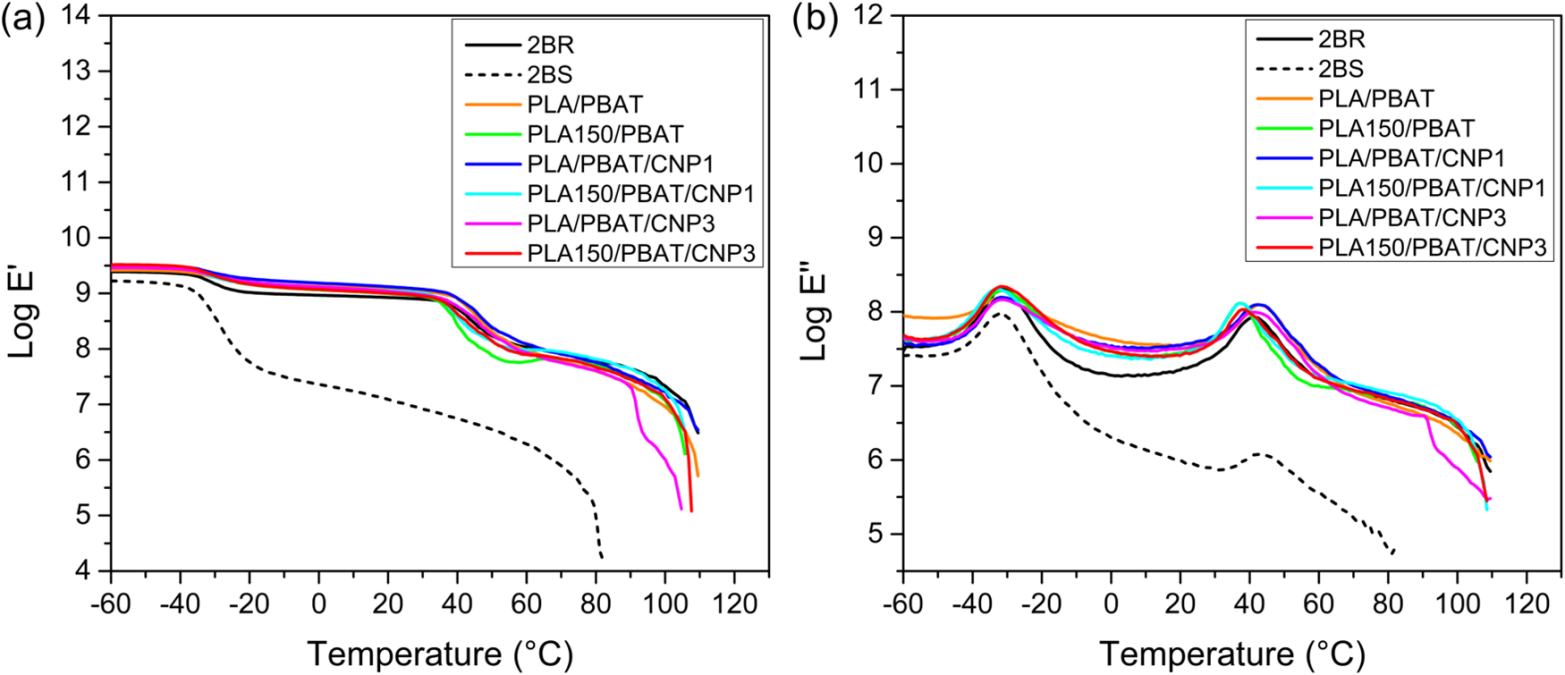
Comparison of 2BR and 2BS model predictions with experimental data for PLA/PBAT blend subjected to a maximum gamma irradiation dose of 150 kGy and reinforced with 1 and 3% CNP for: (a) storage tensile modulus (Log *E*’); (b) loss tensile modulus (Log *E*″).

### 3.4 Differential Scanning Calorimetry (DSC)

The thermal behavior of PLA/PBAT/CNP nanocomposites, with CNP loadings of 1 and 3%, was analyzed using differential scanning calorimetry (DSC). Results are collected in Figure 8 and summarized in Table 4. During the second heating cycle, the glass transition temperature (Tg) of the nanocomposites increased with the addition of CNPs. The values for *T*_g_ from DSC are higher than those observed in DMA analysis. This difference can be attributed to variations in measurement methods and conditions, particularly the heating rates (3 °C/min for DMA vs 10 °C/min for DSC). Higher heating rates in DSC shift *T*_g_ to elevated temperatures due to the time–temperature superposition principle, thermal lag, and heat transfer limitations.^48^

**Figure 8:**
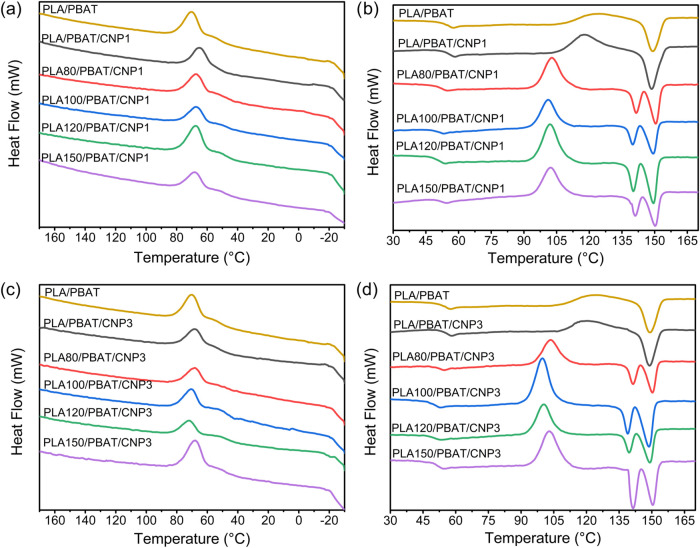
DSC curves during cooling from the melt (a, c) and second heating scan (b, d) for PLA/PBAT blend and PLA/PBAT/CNP nanocomposites with (a, b) 1% of CNP; (c, d) 3% of CNP.

**Table 4:** Thermal Properties of PLA, PBAT, Their Blends and Nanocomposites with Different Gamma Irradiation Doses Obtained by DSC

sample	*T*_g_^a^ (°C)	*T*_hc_^b^ (°C)	Δ*H*_hc_ ^c^ (J/g)	*T*_m1_^d^ (°C)	*T*_m2_^e^ (°C)	Δ*H*_m_ ^f^ (J/g)	*T*_cc_^g^ (°C)	Δ*H*_cc_ ^h^ (J/g)	*X*_cDSC PLA_[Table-fn t4fn9] (%)	*X*_cDSC PBAT_^j^ (%)	*X*_cXRD_^k^ (%)
PLA/PBAT	56.9	115.8	7.6	148.1	-	13.2	76.6	9.9	7.1	5.8	15.9
PLA/PBAT/CNP1	58.1	117.3	12.2	148.4	-	14.8	65.3	9.8	10.5	8.6	27.9
PLA80/PBAT/CNP1	54.5	102.8	14.9	141.3	150.2	15.1	67.3	10.1	10.8	8.8	29.5
PLA100/PBAT/CNP1	53.2	101.2	13.5	139.9	149.2	14.2	67.3	7.9	13.6	11.1	28.5
PLA120/PBAT/CNP1	53.5	101.9	14.2	139.9	149.2	14.3	67.4	13.8	1.1	0.9	28.8
PLA150/PBAT/CNP1	54.5	102.2	13.1	141.0	150.2	13.2	68.1	8.5	10.0	8.1	28.4
PLA/PBAT/CNP3	57.9	119.6	7.5	148.8	-	11.3	68.3	7.1	9.1	7.4	24.7
PLA80/PBAT/CNP3	54.7	103.5	13.9	141.3	150.1	14.1	68.3	8.2	12.8	10.4	24.9
PLA100/PBAT/CNP3	53.2	101.2	13.9	139.9	149.3	14.3	67.3	9.0	11.3	9.2	23.9
PLA120/PBAT/CNP3	53.3	100.5	14.0	139.3	149.0	14.5	72.0	6.6	17.0	13.8	24.3
PLA150/PBAT/CNP3	53.8	102.9	12.8	140.5	150.2	14.5	68.0	13.5	2.1	1.7	23.3

a Glass transition temperature.

b Hot crystallization temperature.

c Hot crystallization enthalpy.

d First peak of melting temperature.

e Second peak of melting temperature.

f Melting enthalpy.

g Cold crystallization temperature.

h Cold crystallization enthalpy.

I Degree of crystallinity from DSC of PLA.

j Degree of crystallinity from DSC of PBAT.

k Degree of crystallinity from XRD.

This increase in *T*_g_ from DSC can be attributed to intermolecular interactions between hydroxyl groups of CNPs and carbonyl groups of PLA and PBAT, which restrict polymer chain mobility and effectively raise *T*_g_. These results are consistent with prior studies by Avella et al.^49^ and Le Digabel and Avérous,^50^ who reported similar effects of nanocellulose on polymer matrices. However, when PLA was subjected to gamma irradiation with increasing doses, a reduction in *T*_g_ was observed. As discussed in a previous study on irradiated PLA/PBAT blends,^18^ gamma irradiation induces chain scission, which reduces the molecular weight of PLA and increases chain mobility, thereby lowering *T*_g_. In this study, the incorporation of cellulose nanoparticles (CNPs) appears to partially counteract this effect. CNPs likely introduce physical cross-linking or hydrogen bonding interactions, which restrict polymer chain mobility and slightly increase *T*_g_. Despite this mitigating effect, the reduction in *T*_g_ caused by irradiation still persists in the nanocomposites, although to a lesser extent compared to unfilled blends.

The melting temperature (*T*_m_) and melting enthalpy (Δ*H*_m_) of the nanocomposites remained relatively unchanged with increasing CNP content. This suggests that CNPs do not significantly alter the overall crystalline structure, or the total amount of crystalline material formed during the second heating cycle. However, upon applying gamma irradiation, a division of the melting peak was observed. This phenomenon suggests that irradiation induces the formation of lower-molar-mass segments within the PLA chains, which tend to melt at lower temperatures compared to the higher-molar-mass chains.^18^ As the dose of gamma irradiation increased, the interval between the two melting peaks also expanded, indicating a greater degree of chain scission and a broader distribution of molecular weights within the material. Meantime, the hot crystallization temperature (*T*_hc_) showed a slight increase, while the hot crystallization enthalpy (Δ*H*_hc_) remained stable. This indicates that CNPs may act as nucleating agents, facilitating the crystallization of PLA and PBAT during heating, as supported by Sarul et al.^24^ The ability of CNPs to promote nucleation is likely due to their high surface area and ability to interact with polymer chains, promoting crystallization.^51^ Additionally, as the dose of gamma irradiation increases, the degree of crystallinity slightly increases, potentially through increased interfacial interactions between PLA and PBAT by affecting the amorphous phase.^18^

Conversely, the cold crystallization temperature (*T*_cc_) decreased, and the cold crystallization enthalpy (Δ*H*_cc_) increased with CNP addition. This behavior suggests that CNPs may hinder the kinetics and extent of PLA/PBAT crystallization during cooling, potentially promoting the formation of less perfect crystals that melt at lower temperatures, as explained by Fukushima et al.^52^ However, the calculated degree of crystallinity (*X*_c_) reveals that the addition of 1% CNP results in a slight increase in crystallinity, while the incorporation of 3% CNP leads to a reduction, though still higher than the neat blend. This suggests an overall improvement in crystallinity due to the nucleating effect of CNPs, which facilitates the formation of crystalline regions.

Therefore, it is evident that gamma irradiation and CNP incorporation have complementary effects on the thermal properties of PLA/PBAT blends. Gamma irradiation reduces the molecular weight of PLA, increasing chain mobility and lowering *T*_g_, while CNPs introduce physical interactions that restrict chain mobility and elevate *T*_g_. Similarly, gamma irradiation enhances crystallization by modifying the amorphous phase, while CNPs promote nucleation and influence crystal morphology, leading to an overall improvement in crystallinity.

### 3.5 Thermogravimetric Analysis (TGA)

The thermal degradation behavior of PLA/PBAT nanocomposites reinforced with 1 and 3% cellulose nanoparticles (CNPs) was investigated using thermogravimetric analysis (TGA) and derivative thermogravimetry (DTG), as illustrated in Figure 9 and summarized in Table 5. Initially, the incorporation of CNPs has been shown to improve the thermal stability of nanocomposites, particularly at higher CNP loadings (3%), as also observed in another study.^53^ This improvement can be attributed to the reinforcing effect of CNPs, which act as barriers to heat and mass transfer during thermal decomposition. The high surface area and polar nature of CNPs likely create strong interactions with the polymer matrix, delaying the onset of degradation and increasing the temperatures corresponding to 5 and 50% weight loss. However, this beneficial effect of CNPs on thermal stability was compromised by gamma irradiation. As the irradiation dose increased, a subsequent reduction in the onset degradation temperature was observed. This reduction is consistent with the findings of our previous study,^18^ where gamma irradiation-induced chain scission in PLA weakened the polymer structure and lowered its thermal stability. In the current study, the detrimental impact of irradiation was evident not only in the onset temperature but also in the temperatures corresponding to 5 and 50% weight loss, further confirming the vulnerability of the PLA matrix to irradiation-induced degradation.

**Figure 9:**
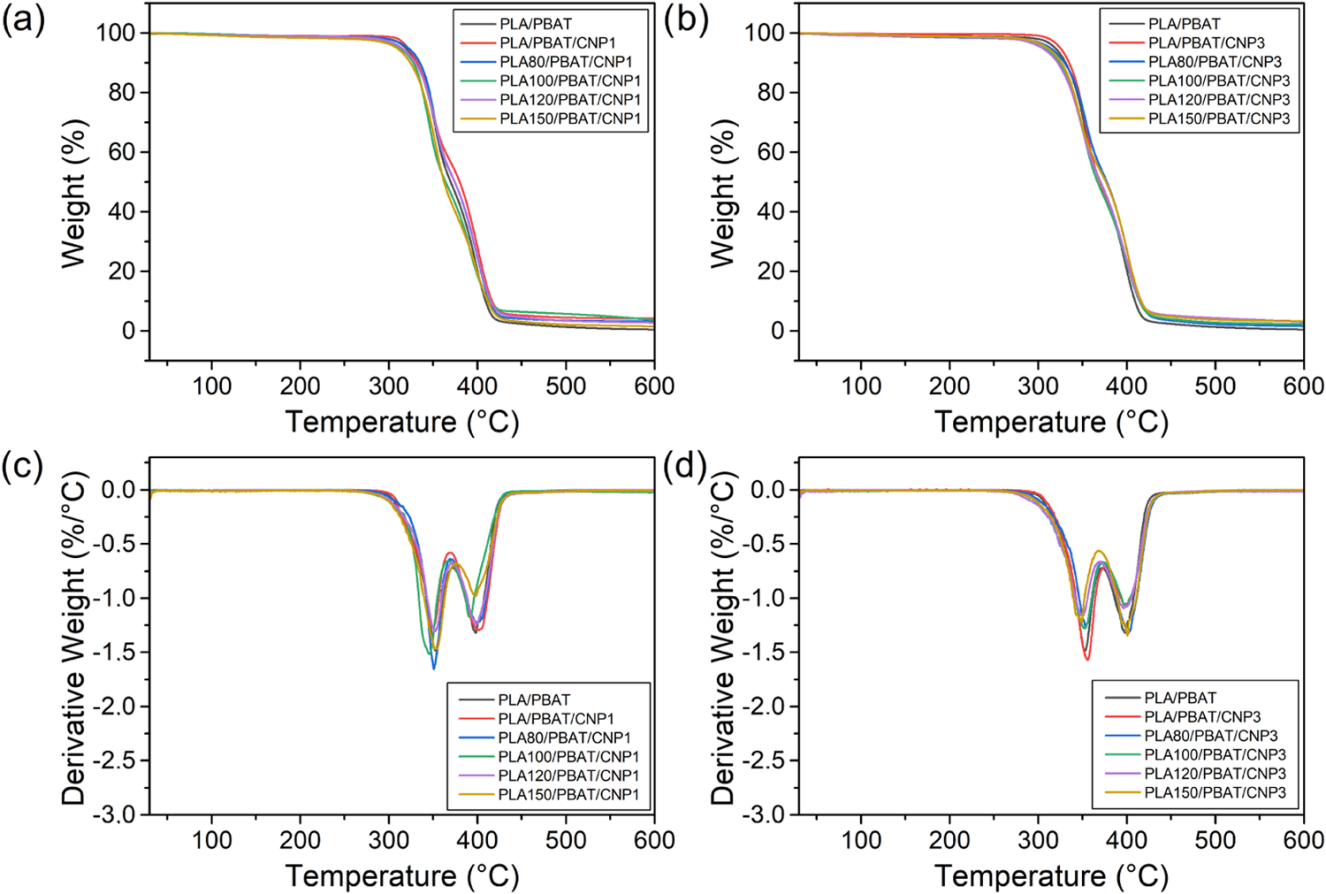
TGA (a, b) and DTG (c, d) curves for PLA/PBAT blend and PLA/PBAT/CNP nanocomposites with different irradiation doses.

**Table 5:** Thermogravimetric Analysis Data for PLA/PBAT Blend and Their Nanocomposites with CNP with Different Gamma Irradiation Doses

sample	*T*_onset_ (°C)	*T*_5%_ (°C)	*T*_50%_ (°C)	*T*_DTG max_ (°C)	*R*_600 °C_ (%)
PLA/PBAT	329.3	319.0	369.8	353.0/398.3	0.4
PLA/PBAT/CNP1	328.9	321.5	380.0	350.0/400.6	4.1
PLA80/PBAT/CNP1	333.7	321.7	374.3	351.1/400.0	3.2
PLA100/PBAT/CNP1	325.2	311.5	364.0	345.8/391.0	3.7
PLA120/PBAT/CNP1	327.7	314.3	374.3	351.7/398.8	2.7
PLA150/PBAT/CNP1	325.8	308.7	362.3	353.6/398.8	1.5
PLA/PBAT/CNP3	332.5	324.3	369.5	355.4/398.2	3.2
PLA80/PBAT/CNP3	327.6	313.5	377.3	353.6/401.3	1.5
PLA100/PBAT/CNP3	321.7	308.7	366.8	351.7/400.0	2.2
PLA120/PBAT/CNP3	322.8	304.3	370.3	350.0/396.4	3.0
PLA150/PBAT/CNP3	322.3	310.3	376.3	349.4/400.6	3.2

Interestingly, the addition of CNPs did not exhibit a significant or consistent trend in the DTG peak temperatures, which represent the maximum rate of degradation. This suggests that while CNPs influence the overall thermal stability of the nanocomposites, their presence does not substantially alter the degradation kinetics of the polymer matrix. However, in the irradiated nanocomposites, a reduction in the intensity of the DTG peak was observed. This also suggests that gamma irradiation alters the degradation behavior, potentially by promoting more extensive chain scission or modifying the polymer’s thermal decomposition pathways.

The residual mass at 600 °C showed a slight increase with increasing CNP content, reflecting the contribution of CNPs to the inorganic or carbonaceous residue left after the organic polymer matrix was decomposed. This result aligns with prior studies that have demonstrated the ability of cellulose-based nanofillers to leave behind charred residues during thermal degradation.^45,54,55^ The presence of CNPs in the nanocomposite structure not only enhances the residual mass but also confirms their successful incorporation into the blend.

Therefore, it is evident that gamma irradiation and CNP incorporation have contrasting effects on the thermal degradation behavior of PLA/PBAT blends. Gamma irradiation reduces the thermal stability of PLA matrix by inducing chain scission, as evidenced by the lower onset and degradation temperatures observed in both neat PLA/PBAT blends and their nanocomposites. Conversely, CNPs enhances thermal stability and increases residual mass, confirming their reinforcing effect and successful integration into the blend. However, the beneficial effects of CNPs are masked by the structural damage caused by gamma irradiation, particularly at higher doses. This highlights the need for optimization when combining these two modification strategies (gamma irradiation and CNP incorporation) to achieve the desired balance between improved compatibility and thermal performance.

### 3.6 Tensile Tests

The mechanical tensile test results for PLA/PBAT nanocomposites with 1 and 3% cellulose nanoparticles were evaluated under varying gamma irradiation doses, as shown in Figure 10 and summarized in Table 6. For neat PLA/PBAT/CNP nanocomposites, the addition of CNPs did not significantly improve tensile strength, likely due to poor dispersion of CNPs within the polymer matrix, also observed in SEM analysis. This observation aligns with findings reported by other researchers,^55^ who emphasize that the reinforcing effect of nanocellulose strongly depends on its uniform dispersion and effective interaction with the polymer matrix. In this study, the lack of improvement in PLA/PBAT irradiated blends suggests that the processing method or intrinsic properties of CNPs have been insufficient to achieve optimal dispersion. Poor dispersion and agglomeration likely compromised interfacial adhesion, which negatively impacts in tensile strength mechanical performance.

**Figure 10:**
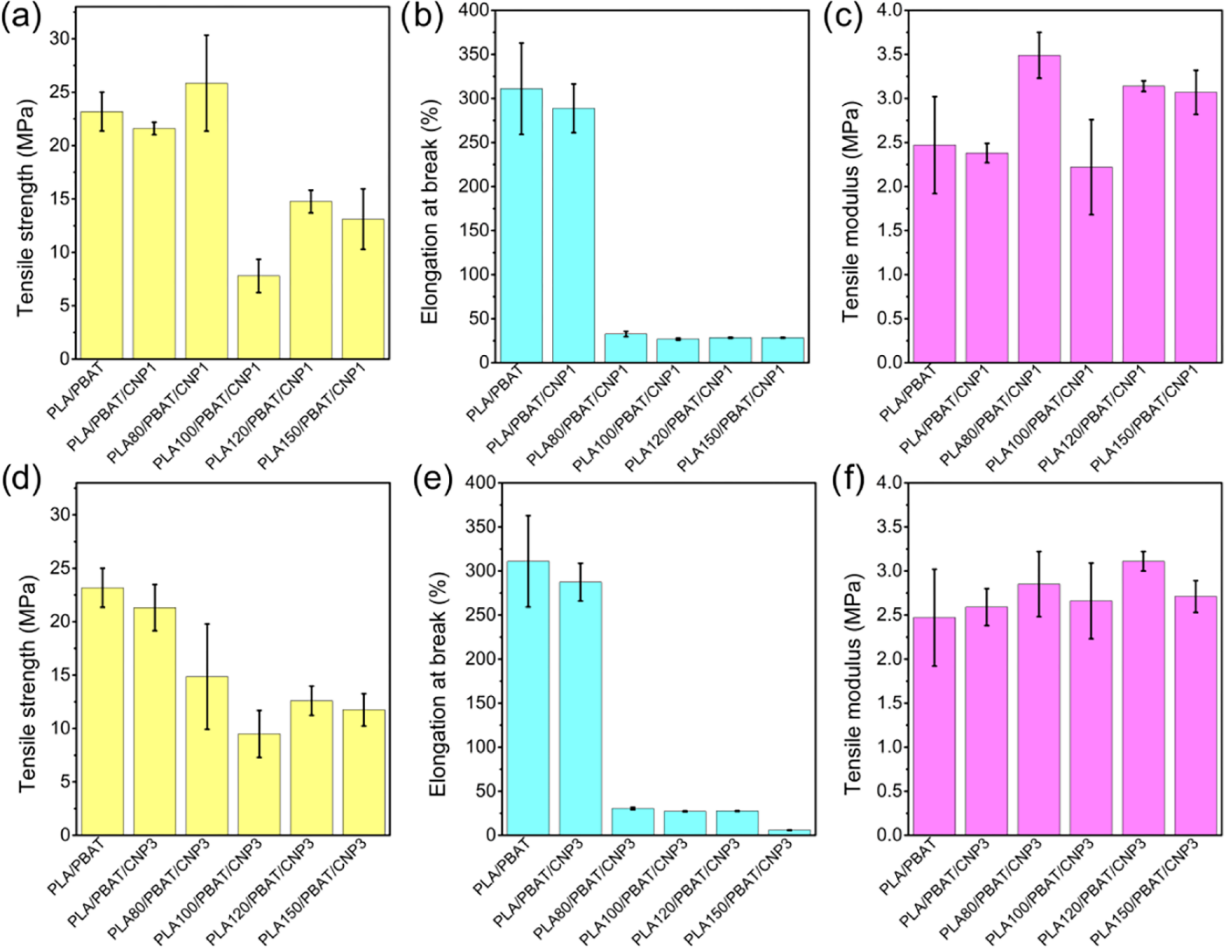
Tensile mechanical properties for PLA/PBAT blend and PLA/PBAT/CNP nanocomposites with different gamma irradiation doses: (a–c) with 1% of CNP; (d–f) with 3% of CNP.

**Table 6:** Tensile Properties of PBAT/PLA Blend and PLA/PBAT/CNP Nanocomposites with and without Gamma Irradiation

sample	tensile strength at yield (MPa)	tensile modulus (MPa)	elongation at yield (%)	tensile stress at break (MPa)	elongation at break (%)
PLA/PBAT	23.18 ± 1.83	2.47 ± 0.55	24.80 ± 12.03	21.91 ± 3.50	310. 98 ± 51.87
PLA/PBAT/CNP1	21.60 ± 0.58	2.38 ± 0.11	26.46 ± 9.99	21.34 ± 0.46	288.72 ± 27.60
PLA80/PBAT/CNP1	25.84 ± 4.50	3.49 ± 0.26	31.53 ± 1.66	22.93 ± 4.62	32.61 ± 3.05
PLA100/PBAT/CNP1	7.8 ± 1.56	2.22 ± 0.54	26.80 ± 1.10	6.78 ± 1.06	26.81 ± 1.10
PLA120/PBAT/CNP1	14.76 ± 1.06	3.14 ± 0.06	28.42 ± 0.61	14.38 ± 1.31	28.43 ± 0.60
PLA150/PBAT/CNP1	13.11 ± 2.83	3.07 ± 0.25	28.38 ± 0.45	13.10 ± 2.84	28.39 ± 0.43
PLA/PBAT/CNP3	21.31 ± 2.16	2.59 ± 0.21	30.36 ± 0.44	21.78 ± 1.07	287.36 ± 21.31
PLA80/PBAT/CNP3	14.85 ± 4.93	2.85 ± 0.37	30.39 ± 1.25	14.75 ± 4.90	30.42 ± 1.26
PLA100/PBAT/CNP3	9.49 ± 2.20	2.66 ± 0.43	27.18 ± 0.54	9.49 ± 2.20	27.20 ± 0.54
PLA120/PBAT/CNP3	12.60 ± 1.37	3.11 ± 0.11	27.56 ± 0.38	12.60 ± 1.37	27.56 ± 0.38
PLA150/PBAT/CNP3	11.75 ± 1.51	2.71 ± 0.18	5.87 ± 0.29	11.71 ± 1.58	5.87 ± 0.29

For irradiated nanocomposites, at low gamma irradiation doses (80 kGy), the incorporation of 1% CNP led to a slight improvement in tensile strength. However, increasing the CNP content to 3% resulted in a reduction in tensile strength, suggesting that higher CNP loadings exacerbate dispersion issues or introduce stress concentration points. At higher irradiation doses (up to 100 kGy), a further decrease in tensile strength was observed. This decline is attributed to the increased fragility of PLA caused by extensive chain scission, as evidenced by SEC analysis. The molecular weight reduction at 100 kGy diminishes the reinforcing effect of CNPs, as the polymer matrix becomes too brittle to benefit from the nanofiller.

Regarding tensile modulus, a slight decrease was observed from neat PLA/PBAT blends to the PLA/PBAT/CNP1 nanocomposite, while a pronounced increase was noted for PLA/PBAT/CNP3, indicating that higher amount of CNPs contribute to the stiffness of the material, likely through physical interactions that restrict polymer chain mobility. As for the irradiated nanocomposites, the application of gamma irradiation further enhanced this effect, potentially by promoting cross-linking or structural reorganization within the polymer matrix.^18^ For irradiated nanocomposites with 3% CNP, the tensile modulus appears to increase subtly as the irradiation dose increases. In contrast, for irradiated nanocomposites with 1% CNP, the tensile modulus shows constant variation with increasing irradiation dose, indicating a less predictable response at lower CNP content. Concerning elongation at break, both 1 and 3% CNP nanocomposites exhibited a small reduction compared to the neat blend. When combined with gamma irradiation, this reduction became more pronounced, likely due to the increased brittleness of the polymer matrix at higher doses. Therefore, while CNPs enhance stiffness, their impact on tensile strength is limited by poor dispersion and brittleness induced by gamma irradiation. These results align with the observed trends in SEC analysis. At 100 kGy, chain scission dominates, resulting in a significant reduction in mechanical performance. At higher doses, structural reorganization occurs, leading to partial recovery of mechanical properties due to the formation of cross-linked or branched structures.

### 3.7 Rheological Properties

The rheological properties of PLA/PBAT nanocomposites reinforced with 1% and 3% of CNP are presented in Figure 11. The results reveal that the incorporation of CNPs does not significantly alter the complex viscosity (η*), storage modulus (*G*′), or loss modulus (*G*″) at low frequencies compared to the neat blend without CNPs. However, a slight reduction in these properties is observed at higher frequencies for both 1 and 3% CNP nanocomposites. Poor dispersion of CNPs within the immiscible PLA/PBAT matrix likely contributes to the lack of improvement in η*, *G*′, and *G*″. Agglomeration of CNPs can create stress concentration points, further reducing the material’s ability to resist deformation. Additionally, the absence of a compatibilizing agent exacerbates the issue by limiting interfacial adhesion between CNPs and the polymer matrix. These factors collectively hinder the reinforcing effect of CNPs, preventing them from effectively enhancing the rheological properties of the nanocomposites. Also, increasing the irradiation dose in the nanocomposites resulted in a further reduction of η*, *G*′, and *G*″, consistent with the behavior of irradiated PLA/PBAT blends at these doses.^18^ This decrease can be attributed to the dominance of chain scission reactions, reducing its molecular weight and viscosity. At 100 kGy, the chain scission events were most pronounced, resulting in a sharp decline in rheological performance. While structural reorganization and potential cross-linking at higher doses (e.g., 120 and 150 kGy) partially recover rheological behavior.

**Figure 11:**
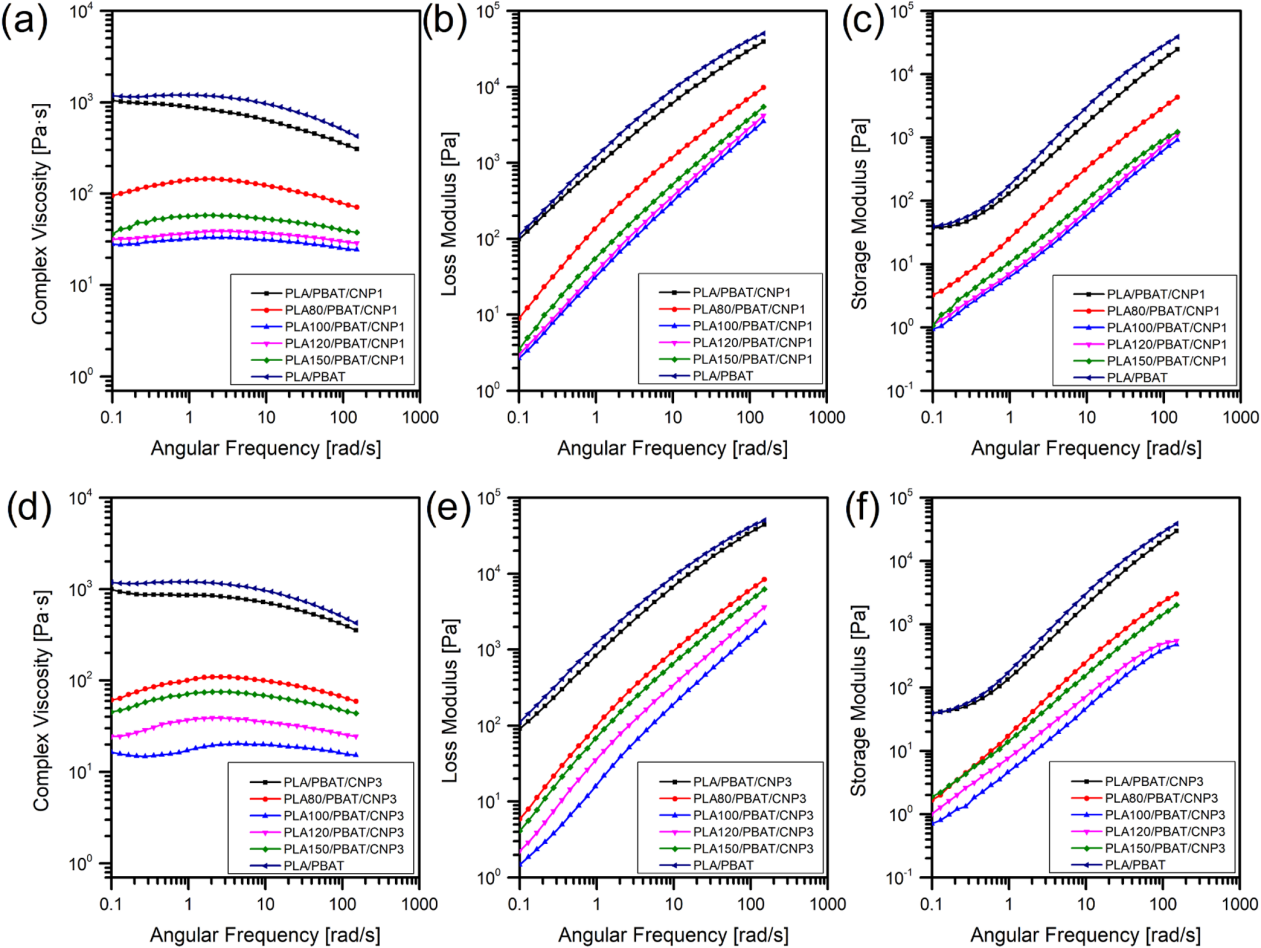
Rheological properties for PLA/PBAT blend and PLA/PBAT/CNP nanocomposites with 1% (a–c) and 3% of CNP (d–f) under different irradiation doses.

While the addition of nanocellulose is generally expected to enhance the rheological properties of polymer matrices, as reported in prior studies,^21,24,56,57^ the current findings diverge from this expectation. Specifically, low nanocellulose loadings such as 1% do not significantly improve the rheological properties of PLA/PBAT blends. At higher loadings (e.g., 3% or 5%), other studies have demonstrated significant increases in η* and *G*′ due to the formation of a percolated nanocellulose network and a transition from liquid-like to solid-like behavior.^24,57^ However, in this study, the observed reduction in rheological properties, even with CNP addition, can be explained by three key factors. First, the chain scission induced by gamma irradiation significantly weakens the PLA matrix, negating the reinforcing effect of CNPs. Second, in the nonirradiated nanocomposites, the inherent immiscibility of the PLA/PBAT matrix, combined with the absence of compatibilizing agent, limits the ability of CNPs to effectively reinforce the blend. As a result, CNP addition does not lead to the expected improvements in rheological behavior, highlighting the challenges associated with achieving uniform dispersion and strong interfacial adhesion in immiscible polymer blends. Third, the irregular shape of CNP particles, along with the absence of long fibers necessary for network formation, may contribute to the reduction in rheological properties. The lack of a percolated network within the CNP-reinforced matrix suggests that the processing conditions or CNP characteristics may not be optimized to achieve effective reinforcement.

Therefore, the rheological properties of PLA/PBAT/CNP nanocomposites are influenced by both CNP content and gamma irradiation dose. While CNPs are expected to enhance η*, *G*′, and *G*″ through network formation and restricted chain mobility, their reinforcing effect is limited in the current study by poor dispersion and the immiscibility of the PLA/PBAT matrix. Gamma irradiation further complicates the behavior of these nanocomposites by inducing chain scission, which weakens the polymer matrix and diminishes its ability to benefit from CNP reinforcement.

### 3.8 Attenuated Total Reflection Fourier-Transform Infrared Spectroscopy (ATR-FTIR)

FTIR spectra for PLA/PBAT nanocomposites reinforced with 1 and 3% of CNP are presented in Figure 12. A slight increase in the band at 3295 cm^–1^, corresponding to O–H stretching vibrations, was observed with the addition of CNPs. This increase indicates a greater presence of hydroxyl groups, which are consistent with the polar nature of cellulose. The incorporation of CNPs introduces additional hydroxyl functionalities into the blend that potentially enhances hydrogen bonding interactions between CNPs and the polymer matrix. In the region between 2968 and 2858 cm^–1^, attributed to the symmetrical and asymmetrical stretching vibrations of −CH groups, a progressive attenuation of the bands was observed with increasing CNP content. This attenuation was more pronounced at 3% CNP and became even more significant with higher irradiation doses. This behavior suggests that gamma irradiation-induced chain scission alters the vibrational environment of the −CH groups, potentially disrupting the regularity of the polymer chains. The characteristic C=O stretching band at 1750 cm^–1^ exhibited an initial increase with 1% CNP but subsequently decreased when 3% CNP was added. Furthermore, as the radiation dose increases, a significant reduction in the intensity of this peak was observed. This trend suggests that while low CNP loadings may promote interactions between CNPs and the polymer matrix, higher CNP contents and gamma irradiation doses disrupt these interactions. The reduction in C=O intensity could be linked to the oxidation and chain scission processes induced by gamma irradiation, which lead to the formation of additional functional groups such as carboxyl and hydroxyl terminals, as explained elsewhere.^18^

**Figure 12:**
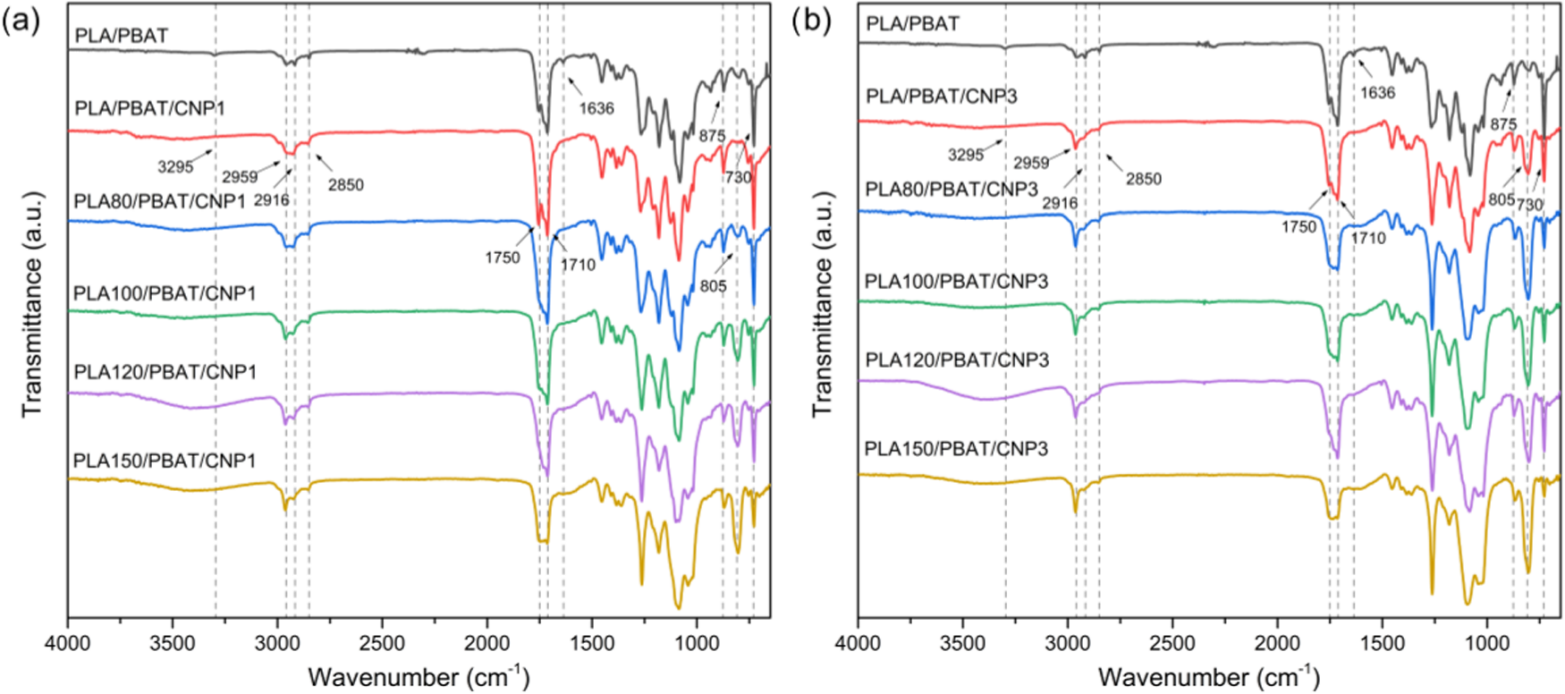
FTIR spectra for PLA/PBAT blend and PLA/PBAT/CNP nanocomposites with different gamma irradiation doses: (a) with 1% of CNP; (b) with 3% of CNP.

Notably, in the C=C stretching vibration band at 1636 cm^–1^, its reduction at higher doses of gamma irradiation was expected as observed in gamma-irradiated PLA/PBAT blends,^18^ because gamma irradiation can induce chain scission followed by recombination reactions, potentially leading to the formation of other functional groups (e.g., carbonyls) instead of C=C double bonds. However, the presence of CNPs makes this peak fully disappear. This disappearance suggests that the presence of CNPs and the concurrent effects of irradiation suppress the formation or stabilization of C=C double bonds within the blend. The bands at 875 and 730 cm^–1^, associated with the out-of-plane bending mode of =C–H groups in the benzene ring of PBAT, showed a tendency to decrease with both 1 and 3% CNP additions. This reduction may indicate a change in the local environment of the aromatic rings, potentially caused by interactions with CNPs or structural rearrangements within the blend. Conversely, the band at 805 cm^–1^, related to C–H in two adjacent hydrogen aromatic rings, showed an increase with 1% CNP and a further increase with 3% CNP, particularly as the irradiation dose was increased. This increase could be attributed to a change in the arrangement or environment of the aromatic rings caused by the presence of nanocellulose^58^ and the chain scission induced by gamma irradiation.

Therefore, the increase in hydroxyl group presence, attenuation of −CH stretching bands, and changes in carbonyl and aromatic vibrations provide evidence of interactions between CNPs and the polymer matrix. Gamma irradiation further modifies the blend system by inducing chain scission and recombination reactions, which alter the chemical environment and promote compatibility.

### 3.9 X-ray Diffraction Analysis (XRD)

XRD patterns for nanocomposites with 1% CNP (Figure 13a) reveal a notable increase in the intensity of peaks at approximately 2θ = 16.1 and 17.4°, which are characteristic of the crystalline regions of PLA and PBAT, respectively. This increase suggests a slight improvement in the degree of crystallinity of the matrix, also observed in Table 4, likely due to the nucleating effect of CNPs. These changes suggest good interactions between the PBAT/PLA blend and CNP, potentially contributing to the observed crystallinity variations.^25^ The amorphous and crystalline regions of nanocellulose likely interact with the corresponding regions of the PLA/PBAT blend.^57^

**Figure 13:**
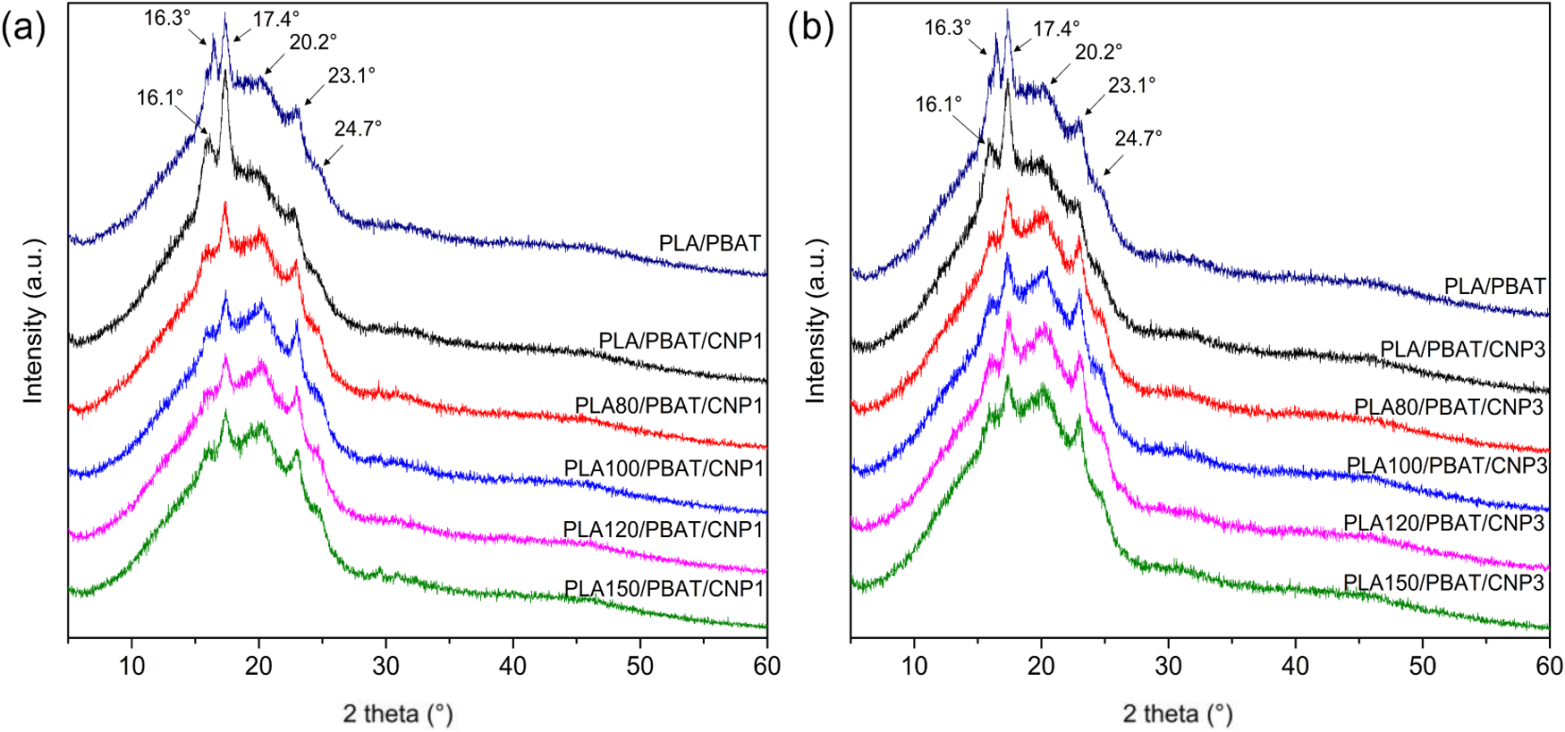
XRD patterns for PLA/PBAT blend and PLA/PBAT/CNP nanocomposites with different absorbed irradiation doses: (a) with 1% of CNP; (b) with 3% of CNP.

For nanocomposites with 3% CNP (Figure 13b), a minor reduction in crystallinity was observed compared to 1% CNP, although the overall crystallinity remained higher than that of the neat blend without CNP. Notably, only the peak at 2θ = 17.4° showed an increase in intensity, while other peaks exhibited less pronounced changes. This behavior suggests that higher CNP loadings may lead to agglomeration or poor dispersion, which could hinder the uniform nucleation of crystalline domains. The presence of CNPs likely enhances these interactions, promoting partial crystallization but also introducing complexities at higher loadings.

Consistent with the trends observed for PLA/PBAT blends,^18^ the nanocomposites containing PLA irradiated at 80 kGy exhibited the highest degree of crystallinity. This observation is visually evidenced from the increased intensities of the peaks at 2θ = 16.1 and 17.4°. However, as the irradiation dose increased beyond 80 kGy, a slight decrease in crystallinity was observed, indicated by the reduction in peak intensities at 16.1 and 17.4° and a concurrent increase in the peaks at 2θ = 20.6 and 23.2°. This trend can be attributed to the competing effects of chain scission and structural reorganization induced by gamma irradiation. At lower doses, chain scission reduces intramolecular stress in the amorphous regions, enhancing chain mobility and promoting crystallization.^18^ However, at higher doses, cross-linking or recombination limit the organization into crystalline structures. Additionally, the increased brittleness of the polymer matrix at higher irradiation doses could disrupt the stability of crystalline domains, further reducing crystallinity.

XRD results are consistent with the thermal analysis findings, where gamma irradiation and CNP incorporation were shown to influence the crystallization behavior of PLA/PBAT blends. DSC analysis revealed that gamma irradiation increases the degree of crystallinity by enhancing chain mobility and reducing intramolecular stress in the amorphous regions. Similarly, the nucleating effect of CNPs promotes crystallization and enhances the crystallinity of the nanocomposites. However, the nonlinear trends observed in XRD analysis, particularly the reduction in crystallinity at higher CNP loadings and irradiation doses, highlight the complexity of these interactions. Poor dispersion of CNPs or cross-linking/recombination at higher doses may disrupt the formation of ordered crystalline structures, leading to a decrease in crystallinity.

### 3.10 Contact Angle Measurements (CA)

Contact angle measurements, as shown in Figure 14, were conducted to evaluate the surface hydrophilicity of PBAT, PLA, PLA films subjected to varying gamma irradiation doses, their corresponding blends, and nanocomposites containing 1 and 3 wt % of CNP. The contact angle serves as a key indicator of surface wettability, with values below 90° indicating hydrophilic (water-attracting) behavior and values above 90° suggesting hydrophobic (water-repelling) characteristics.^59^ Based on this principle, all tested samples exhibited hydrophilic properties, reflecting their affinity for water. However, distinct trends emerged across different material groups. For neat PLA and irradiated PLA films, a progressive decrease in contact angle, from approximately 87 to 77°, was observed with increasing gamma irradiation dose. This reduction signifies a shift toward enhanced hydrophilicity, which can be attributed to the chain scission induced by gamma irradiation. Chain scission likely generates additional hydroxyl end groups on the PLA surface, increasing the material’s polarity and promoting stronger interactions with water.

**Figure 14:**
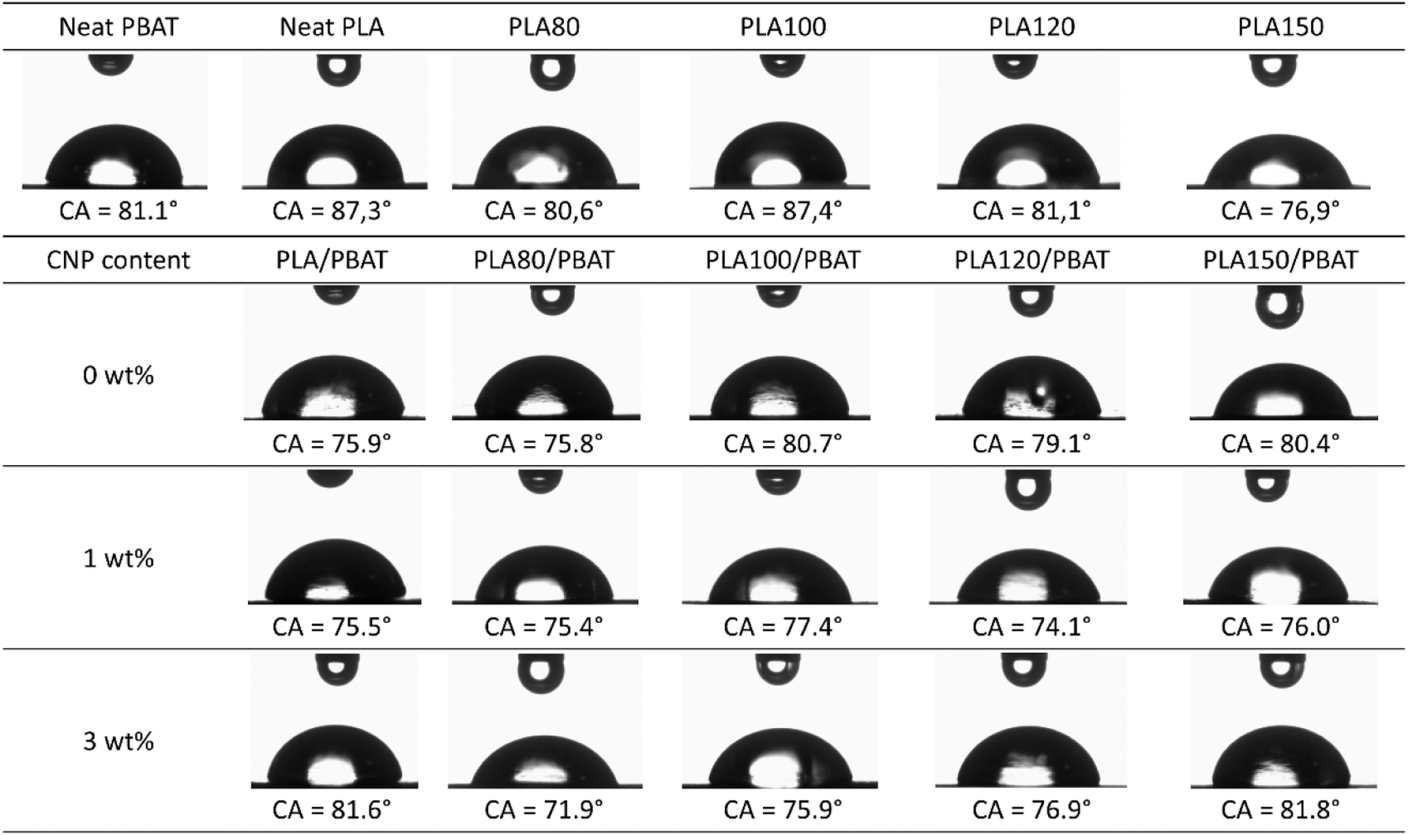
Water contact angle measurements for neat PBAT, neat PLA, PLA irradiated at different doses, their blends and their nanocomposites with 1 and 3% of CNP.

In contrast, PLA/PBAT blends displayed a different behavior. A slight increase in contact angle, from 76 to 80°, was noted with increasing irradiation dose, suggesting a potential reduction in hydrophilicity. This behavior could be due to the combined effects of PLA chain scission and the more hydrophilic nature of PBAT compared to PLA. Gamma irradiation-induced chain scission in PLA could change the proportion of PBAT phase at the blend surface, thereby changing the overall hydrophilicity of the material.

The incorporation of CNPs further modulated the hydrophilicity of the irradiated blends. Notably, the addition of 1 and 3 wt % CNP resulted in a decrease in contact angle for the irradiated blends at lower doses, indicating enhanced hydrophilicity. This effect is likely due to the synergistic interaction between the hydroxyl-rich surface of CNPs and the irradiation-induced modifications in PLA. The presence of CNPs appears to promote a more hydrophilic surface, potentially through hydrogen bonding or other polar interactions. In contrast, nonirradiated blends did not exhibit a similar decrease in contact angle upon CNP addition. This suggests that intact PLA chains in nonirradiated blends combined with low miscibility with PBAT may hinder optimal interactions between CNPs and the polymer matrix, limiting their ability to influence surface hydrophilicity.

Therefore, the contact angle analysis reveals a complex trend between the surface chemistry of PLA, PBAT, and CNPs, as well as the effects of gamma irradiation. Irradiated PLA films demonstrated increased hydrophilicity due to chain scission, while the trends in the blends were more intricate, reflecting the combined influence of both PLA and PBAT on surface properties. The addition of CNPs further highlighted their potential to enhance the hydrophilicity of irradiated PLA/PBAT blends, highlighting the importance of optimizing material composition and processing conditions to achieve the desired surface characteristics.

### 3.11 Biodegradation Test

The weight change of the samples during a six-month (180-day) biodegradation test is illustrated in Figure 15. An initial increase in weight observed during the first few months can be attributed to water absorption from the soil, consistent with the hydrophilic nature of all samples as confirmed by contact angle measurements. Among the materials tested, PLA exhibited a significantly slower degradation profile compared to PBAT. This finding aligns with prior research,^60^ which attributes PLA’s lower biodegradability in soil due to its high glass transition temperature (*T*_g_) of approximately 60 °C, making it less accessible to microbial attack under ambient conditions. However, for irradiated PLA, a clear trend emerged in the later stages of the test: higher irradiation doses resulted in greater weight loss. This observation is supported by Benyathiar et al.,^61^ who demonstrated that gamma irradiation-induced chain scission reduces PLA’s molecular weight. Their study also highlighted that free radicals generated during irradiation can alter PLA’s chemical structure, enhancing its susceptibility to biodegradation through increased mineralization.

**Figure 15:**
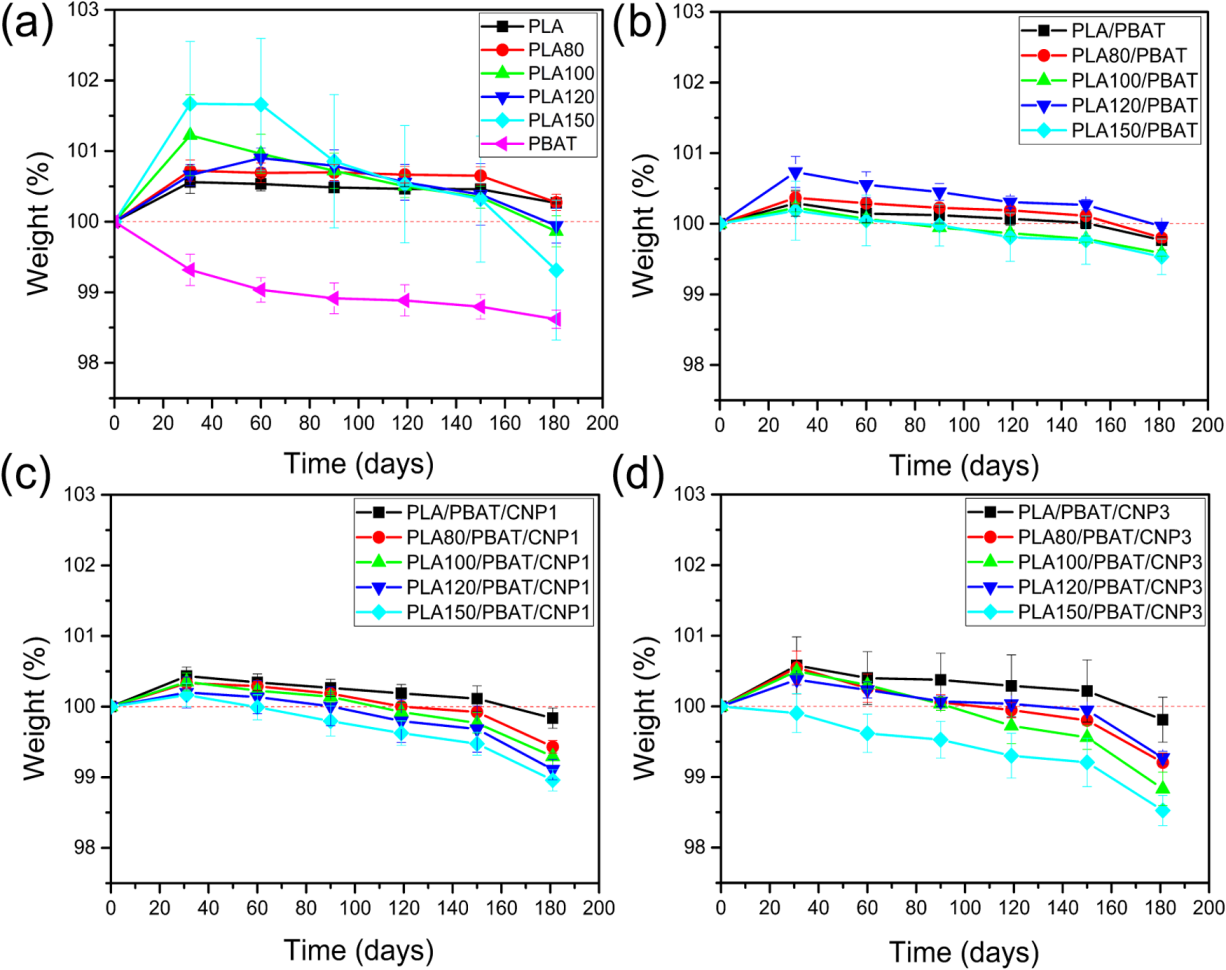
Weight change during biodegradation test: (a) for PLA, PLAs irradiated and PBAT; (b) for PLA/PBAT blends; (c) for PLA/PBAT/CNP1 nanocomposites; (d) for PLA/PBAT/CNP3 nanocomposites.

In PLA/PBAT blends, a direct correlation was observed between the irradiation dose applied to PLA and the extent of weight loss during biodegradation. As the irradiation dose increased, a corresponding increase in weight loss was noted for the blends. This suggests that the chain scission induced by gamma irradiation weakens the polymer structure, thereby facilitating the degradation of the PLA/PBAT blend. Notably, the biodegradation behavior of the blends fell between that of pure PLA and PBAT, reflecting the intermediate degradation rates typical of composite materials. In such systems, the overall degradation rate is influenced by the individual degradation characteristics of the constituent polymers.

The incorporation of cellulose nanoparticles significantly accelerated the biodegradation rate of the blends. The addition of 1% CNP resulted in a noticeable increase in weight loss, and this effect was further amplified with the addition of 3% CNP. This phenomenon can be attributed to the inherent biodegradability of cellulose, which is highly susceptible to microbial degradation. Specifically, the amorphous regions of cellulose provide accessible sites for microbial invasion and enzymatic breakdown, thereby promoting the degradation of the entire Bioplastic matrix.^62^

Despite these variations, the overall weight loss remained below 2% after six months. This limited biodegradation is likely due to the specific test conditions, which simulated microbiological susceptibility in a natural controlled soil environment (23 ± 1 °C, pH 6.5–7.5). It is well-established that the biodegradation of PLA and PBAT is significantly faster under industrial composting conditions, where higher temperatures (50–70 °C) and abundant organic matter accelerate microbial activity.^63^ Thus, the relatively mild conditions of the current study may have restricted the degradation process.

Visual inspection after six months revealed minimal changes in the structural integrity of PLA and irradiated PLA samples (Figure 16). In contrast, PBAT samples exhibited noticeable yellow spots, indicative of potential chemical or biological alterations. Challenges were encountered during the preparation of irradiated PLA disc samples, as the inherent brittleness and altered melt flow properties of irradiated PLA led to discs with nonuniform edges and small openings along the borders. Despite these imperfections, the structural integrity of the irradiated PLA discs remained intact throughout the six-month biodegradation study.

**Figure 16:**
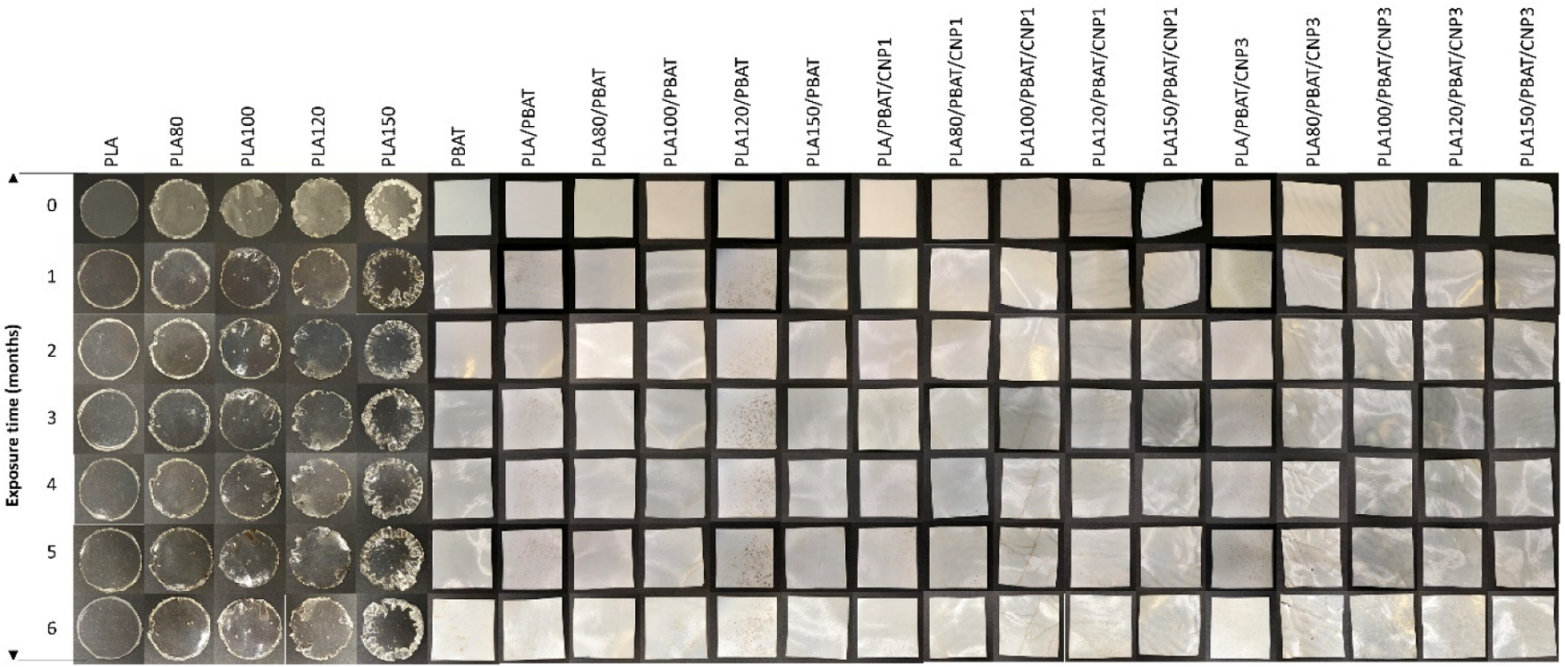
Photographs collected each month of biodegraded samples of PLA, PBAT, PLA/PBAT blend and PLA/PBAT/CNP nanocomposites irradiated at different doses.

For PLA/PBAT blends subjected to varying irradiation doses, visual inspection revealed the presence of brown spots in certain formulations. These spots were most pronounced for nonirradiated PLA/PBAT blend and PLA120/PBAT blend (120 kGy irradiation). PLA80/PBAT blend appeared visually unchanged, while PLA100/PBAT blend exhibited fold marks, which became increasingly prominent over time. Conversely, PLA150/PBAT blend showed no significant visual alterations. These observations suggest that the visual changes, specifically the brown spots and fold marks, are not solely attributable to the irradiation dose. Instead, they appear to be more closely related to the inherent characteristics of the PLA/PBAT blend itself, potentially arising from localized degradation, chemical reactions, or phase separation within the blend.

Finally, for PLA/PBAT/CNP nanocomposites with 1% CNP images revealed distinct differences in appearance based on irradiation dose. The nonirradiated nanocomposite maintained its integrity throughout the six-month observation period, showing minimal visual changes. However, as the irradiation dose increased, noticeable cracks and surface waves began to appear on the film samples. Additionally, a gradual increase in transparency was observed in the irradiated samples over time, suggesting potential thinning or structural alteration of the material. This trend was even more pronounced in the nanocomposites containing 3% CNP. The irradiated samples at this higher CNP concentration exhibited a more rapid progression of cracks and waves, and the increase in transparency was more evident. These visual changes indicate that gamma irradiation induces structural modifications in the nanocomposite matrix, potentially leading to increased brittleness and accelerated degradation. The higher CNP content appears to exacerbate these effects, possibly due to a synergistic interaction between irradiation-induced damage and distribution of CNPs within the matrix.

## 4 Conclusions

This article investigates the effects of incorporating cellulose nanoparticles (CNPs) and gamma irradiation on the properties of poly­(lactic acid) (PLA)/poly­(butylene adipate-co-terephthalate) (PBAT) blends, with focus on addressing the poor mechanical properties observed in highly gamma irradiated PLA/PBAT blends. The findings reveal that while CNPs introduced some improvements in thermal stability, crystallinity, hydrophilicity and biodegradability, their ability to enhance mechanical properties was severely limited by the high degradation caused by gamma irradiation at elevated doses. Gamma irradiation was found to significantly alter the molecular weight distribution and thermal stability of PLA/PBAT blends, primarily through chain scission and partial recombination processes. SEC analysis revealed a pronounced reduction in molecular weight at higher irradiation doses, particularly at 100 kGy, which is associated with extensive chain scission. This effect was accompanied by a decrease in glass transition temperature (*T*_g_) and thermal stability, as confirmed by DSC, DMA and TGA results. However, the incorporation of CNPs partially mitigated these effects by introducing physical interactions, such as hydrogen bonding, which restricted polymer chain mobility and slightly increased *T*_g_. Despite this, the reinforcing impact of CNPs was limited by poor dispersion and agglomeration within the immiscible PLA/PBAT matrix, as evidenced by SEM and rheological analyses.

The mechanical and rheological properties of the nanocomposites were also significantly influenced by CNP addition and gamma irradiation. While low CNP loadings (1%) showed a slight improvement in tensile modulus, higher loadings (3%) exacerbated dispersion issues, leading to reduced tensile strength, elongation at break, and rheological performance. Rheological testing further demonstrated that CNPs did not significantly enhance complex viscosity or storage modulus, likely due to their irregular shape and lack of long fibers necessary for network formation. Interestingly, X-ray diffraction (XRD) and FTIR analyses revealed that CNPs acted as nucleating agents, promoting crystallization and altering the chemical environment of the blend. Finally, the study explored the surface hydrophilicity and biodegradability, which indicated that gamma irradiation increased the hydrophilicity of PLA and its blends, while CNP incorporation further enhanced this effect in irradiated samples. Biodegradation tests conducted over six months showed that irradiation and CNPs accelerated weight loss, with higher CNP concentrations leading to faster degradation.

The novelty of this work lies in evaluating gamma irradiation and CNPs as compatibilization strategies for PLA/PBAT blends, revealing their potential to enhance hydrophilicity, biodegradability, and crystallinity, while identifying key limitations for future optimization. Higher irradiation doses (e.g., 100 kGy) significantly reduced mechanical properties due to chain scission in the PLA matrix, suggesting the need to explore intermediate doses for better compatibility and performance. Additionally, poor CNP dispersion in the PLA/PBAT matrix, especially at higher loadings, caused agglomeration and performance issues. Future studies could address this by investigating cellulose nanofibrils (CNFs), which may form a more effective percolated network to enhance mechanical properties.

## Supplementary Material



## Notes

Additional experiment of atomic force microscopy (AFM) to evaluate the morphology of the CNP obtained after 196 h of ball milling (PDF)
