# Supplementary material for: Impact of High-Dose Gamma Irradiation on PLA/PBAT Blends Reinforced with Cellulose Nanoparticles from Pineapple Leaves
Source: ACS Omega. 2025 Aug 15;10(33):38182–202. doi: 10.1021/acsomega.5c06115 (PMC12391937; doi:10.1021/acsomega.5c06115)

Impact of high-dose gamma irradiation on PLA/PBAT blends reinforced with cellulose nanoparticles from pineapple leaves

Fernanda Andrade Tigre da Costa[ab]*, Alain Dufresne[b] and Duclerc Fernandes Parra[a]

[a]*Universidade de São Paulo, Instituto de Pesquisas Energéticas e Nucleares, IPEN–CNEN/SP. Av. Prof. Lineu Prestes, 2242 – Cidade Universitária, São Paulo, SP, BR 05508-900*

[b]*Universite Grenoble Alpes, Grenoble INP Pagora (LGP2). 461 Rue de la Papeterie, Gières, Auvergne-Rhône-Alpes, FR 38610*

*Fernanda Andrade Tigre da Costa, fernanda.tigre@outlook.com, Department of chemistry and environment (CEQMA) in Nuclear and Energy Research Institute, IPEN–CNEN/SP. Address: Av. Prof. Lineu Prestes, 2242 - Butantã, São Paulo - SP, Brazil, 05508-000.

Supporting Information

Atomic force microscopy was employed to examine the morphology of the CNP obtained after 196 hours of ball milling. The measurements were carried out using a Multimodal AFM (Dimension Icon, Veeco/Bruker, Germany) operating in tapping mode. For sample preparation, a droplet of 0.02 wt% CNP suspension was deposited onto a mica substrate and allowed to dry at room temperature. The resulting AFM images were analyzed using NanoScope Analysis software version 1.40 to determine the dimensions of the fibers. Figure S1 presents the AFM results for the CNP samples, revealing particles with a low aspect ratio and evidence of some aggregation. These observations provide additional comprehensions into the structural characteristics of the CNPs, complementing the SEM analysis discussed in the main manuscript.

Figure S1 - AFM images for CNP samples at different magnifications.

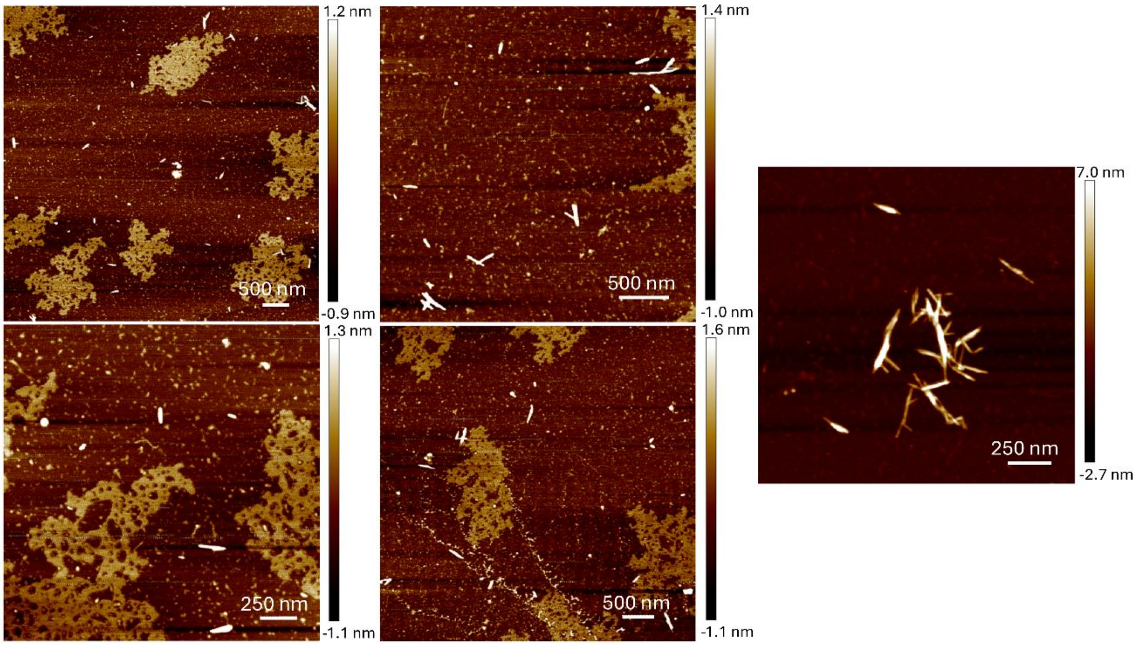

Supplement: Supplementary file 1 [file ao5c06115_si_001.pdf]
